# Comparative genomic analysis of North American *Bradyrhizobium* symbionts of *Chamaecrista fasciculata* (Caesalpinioideae) with related bradyrhizobia

**DOI:** 10.1128/spectrum.00798-26

**Published:** 2026-08-19

**Authors:** Nisha Tak, Zachary Zawada, Marta Maluk, Chrizelle Beukes, Laura Ketelboeter, Hukam S. Gehlot, Prasad Gyaneshwar, Euan K. James

**Affiliations:** 1 Department of Botany, Jai Narain Vyas University 208293, Jodhpur, Rajasthan, India; 2 Department of Biological Sciences, University of Wisconsin–Milwaukee, Milwaukee, Wisconsin, USA; 3 The James Hutton Institute 15554, Dundee, United Kingdom; Connecticut Agricultural Experiment Station, New Haven, Connecticut, USA

**Keywords:** *Chamaecrista fasciculata*, *Bradyrhizobium*, multi-locus sequence analysis, genomic analysis, root nodule, nitrogen fixation

## Abstract

The symbionts of *Chamaecrista fasciculata*, a caesalpinioid legume, were isolated from plants growing in native soil or by host trapping. To these, we added strains from the United States Department of Agriculture and strains isolated earlier from nodules of *C. fasciculata* and *Chamaecrista nictitans*. The phylogeny of 16 strains was determined by examining and comparing sequences of their *rrs* (16S rRNA) genes, the intergenic transcribed spacer region, two “core” housekeeping genes (*recA* and *dnaK*), and two symbiotic (*nodA* and *nifH*) genes. Seven strains were whole-genome sequenced and compared phylogenomically to related strains isolated from Caesalpinioideae nodules. All the symbionts belonged to *Bradyrhizobium* spp. dispersed in both Mega Clades-I and II, with some forming novel clusters and others sharing similarities with previously reported *Chamaecrista* symbionts. All strains nodulated and enhanced *C. fasciculata* growth in nitrogen-free axenic conditions. The draft genomes of seven *Chamaecrista* strains were sequenced, including four isolated from *C. fasciculata* in the United States (CFUW1, CFUSD1, CFUMN1, and USDA3010) and three from Brazil (JHI2701, JHI2711, and Cens1A). Comparative genomic analysis based on average nucleotide identity and digital DNA-DNA hybridization suggested that six strains were closely related to *Bradyrhizobium frederickii*, *Bradyrhizobium ferriligni*, *Bradyrhizobium elkanii*, *Bradyrhizobium australafricanum*, “*Bradyrhizobium brasilense,*” and *Bradyrhizobium yuanmingense*. Meanwhile, the *C. fasciculata* strain USDA3010 and the non-mimosoid Caesalpinioideae *Dimorphandra wilsonii* strain DW12.5 are potential new species.

**IMPORTANCE:**

The legume-rhizobium symbiosis is of immense economic and ecological importance, but most knowledge about its evolution and molecular biology has been obtained through the intense study of only two to three “model” species in the subfamily Papilionoideae, while nodulating symbioses in the sister subfamily, the Caesalpinioideae, which have a separate but parallel evolutionary history, are less well understood. *Chamaecrista fasciculata* appears to be exclusively nodulated by *Bradyrhizobium*, particularly *Bradyrhizobium frederickii*. We propose the symbiosis between *C. fasciculata* and *B. frederickii* CFUW1 as a model system to examine the parallel evolution (to the Papilionoideae) of symbiotic nodulation in the Caesalpinioideae.

## INTRODUCTION

Plants belonging to the Leguminosae (Fabaceae) family are one of the major contributors to the global nitrogen (N) economy through their symbiotic biological N_2_ fixation (BNF) in agricultural and natural environments. Most legumes form symbiotic associations with diazotrophic soil bacteria, collectively known as rhizobia, resulting in the formation of specialized structures on their roots (and occasionally stems) called nodules in which the differentiated rhizobia are accommodated in high numbers and fix N_2_ for the plant in exchange for carbon from photosynthesis supplied by the host plant (1, 2). Extensive studies on the model legumes *Medicago truncatula* and *Lotus japonicus*, both belonging to the subfamily Papilionoideae, have led to substantial advances in determining the evolution and mechanisms of nodulation and symbiotic BNF (3–6). However, a better understanding of BNF in legumes requires extending research beyond the subfamily Papilionoideae (1, 7). Of the six legume subfamilies, only the largest two, the Papilionoideae and the Caesalpinioideae, contain nodulating genera (1, 6, 8). The subfamily Caesalpinioideae diverged from the Papilionoideae nearly 60 million years ago (9, 10), but unlike the Papilionoideae, which is predominantly nodulated, nodulation in the Caesalpinioideae is rare, with the exception of the large tribe Mimosae (formerly the subfamily Mimosoideae), in which nodulation is present in >90% of genera (11). The Caesalpinioideae comprises 23% of legumes, but unlike its sister subfamily, the Papilionoideae, it has no established model system, which, given that it arose simultaneously along with the other five legume subfamilies (10), is a significant gap in our knowledge about how legume nodulation evolved. It is, therefore, possible that nodulation evolved separately in the two nodulating subfamilies, and hence, the widely studied Papilionoid models, *Lotus* and *Medicago* (5), cannot also be assumed to be representative of nodulation in the Caesalpinioids (11).

Nodulation is widespread (and probably a generic feature) in the non-mimosoid Caesalpinioid genus *Chamaecrista* (12); this unusually large genus, comprising more than 360 species (13, 14), has its main center of radiation in Brazil (268 species) with a secondary center in Mexico plus a few herbaceous species that are native to the United States, including the closely related *Chamaecrista fasciculata* and *Chamaecrista nictitans*. Interestingly, this largely New World genus also has some native representatives in Africa (14, 15) and India (16). It was earlier suggested that *Chamaecrista* nodulation may have evolved independently not only from the Papilionoideae but even from the mimosoids, even though the latter are now incorporated into the Caesalpinioideae subfamily (1, 9).

The rhizobial symbionts of *Chamaecrista* spp. are mainly *Bradyrhizobium* in their main center of radiation, Brazil (12, 17, 18), but also in Africa (19, 20), India (16), and the USA (21–26). They are also occasionally nodulated by *Sinorhizobium* (*Ensifer*) in India (16) and rarely by *Paraburkholderia* in Brazil (12). *Chamaecrista fasciculata* was suggested as a model for rhizobial interactions with Caesalpinioideae (7). Given that the *C. fasciculata* genome has been sequenced (27), there is thus a need for a compatible rhizobial model to complement *C. fasciculata* as a model system, but its rhizobial symbionts are only recently beginning to be characterized. For example, the symbionts of *C. fasciculata* growing in the Midwestern United States were characterized as *Bradyrhizobium niftali* (25) and *Bradyrhizobium frederickii* (26), while further strains belonging to *Bradyrhizobium* spp. were found to nodulate *C. fasciculata* in the Mississippi delta (24, 28). Therefore, in this study, we examine a range of strains that were isolated from *C. fasciculata* in the United States and compare them with the few that are already whole-genome sequenced, as well as with strains from other *Chamaecrista* species and other nodulating non-mimosoid Caesalpinioideae species in Brazil and elsewhere.

## MATERIALS AND METHODS

### Bacterial isolation from nodules of field-grown and soil trap host *Chamaecrista fasciculata* plants

**Fig 1:**
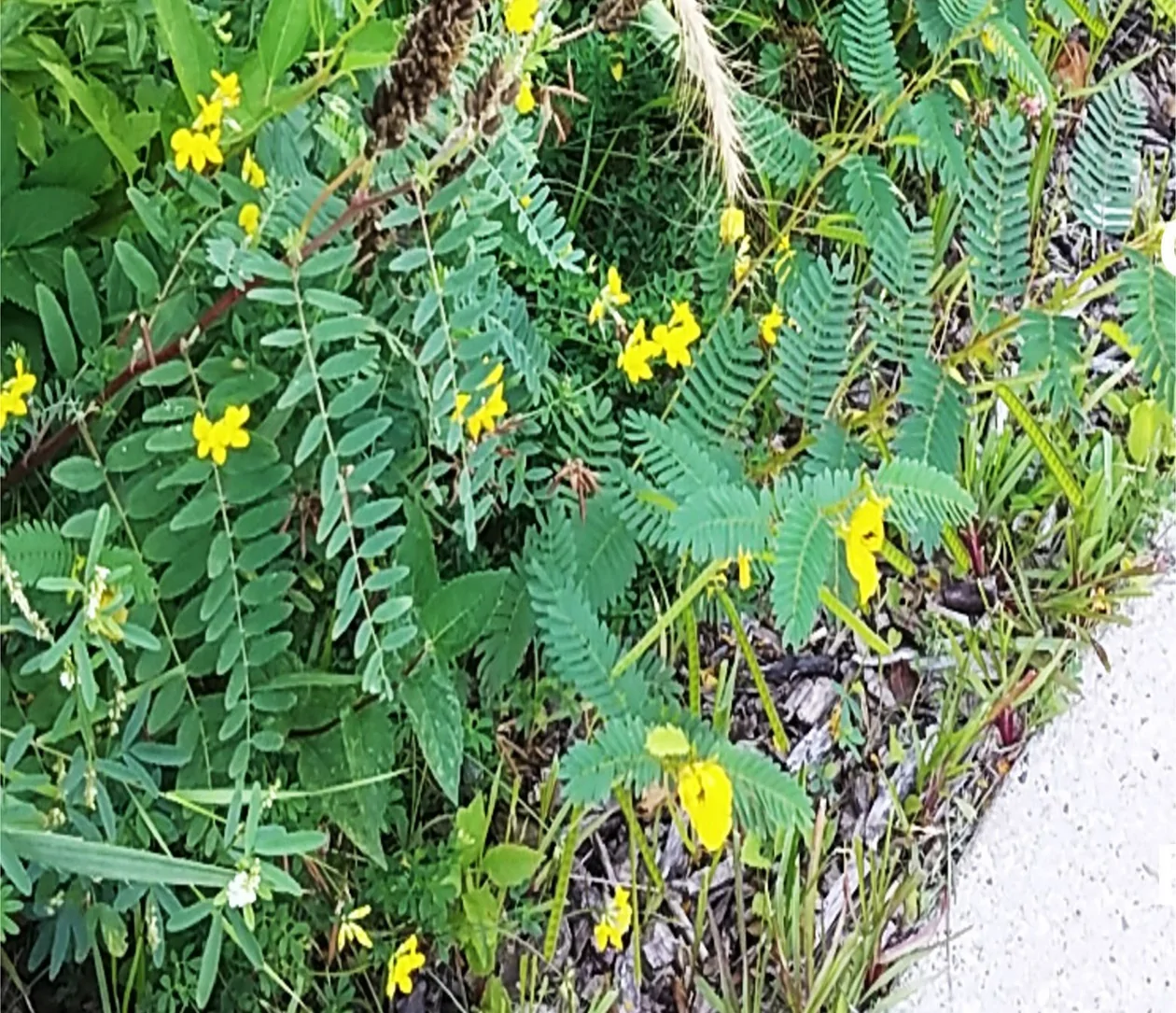
*Chamaecrista fasciculata* plants (in flowering stage) growing at the University of Wisconsin Milwaukee.

*Chamaecrista fasciculata* plants (Fig. 1) growing in the wild at the campus of the University of Wisconsin-Milwaukee, Wisconsin (WI), USA, were sampled, and nodules were harvested. For host-trap experiments, *C. fasciculata* seeds were scarified with concentrated sulfuric acid for 5 min, washed with sterile water, and germinated on sterile moist filter paper at 26°C. Seedlings free of visible contamination were inoculated with soils collected from various states of the United States (Table 1). Uninoculated seedlings were used as a control. At least three seedlings were used for each soil. The seedlings were grown in a growth chamber at 26°C with a 14/10 h light/dark cycle. Nodules were sampled at 30 days after inoculation (dai). Two to three nodules were sampled from each plant, and one isolate per nodule was further purified using yeast-mannitol (YM) agar plates containing Congo red (29). The isolates were grouped based on their location of origin and further selected using their colony morphology on YM plates. The selected isolates were then tested for their ability to nodulate *C. fasciculata* under axenic conditions as follows: seeds were surface sterilized and germinated on sterile and moist filter paper; seedlings free of contamination were then transferred to paper cups containing sterile vermiculite and inoculated and grown in a growth chamber. Plants were harvested at 30 dai and inspected for the formation of nodules. Symbiotic nitrogen fixation was determined by comparing plant growth (dry weight) to uninoculated plants. The nodulating strains from each location were then used for molecular and phylogenetic characterization by multi-locus sequence analysis (MLSA) of selected housekeeping and symbiotic genes.

**TABLE 1:** Origin of strains from this study and reference strains used for comparative analysis^*a*^^,*b*^

S. no.	Strain name	Earlier/other name	Isolation of strains	Site of isolation/reference	Nodulating host
1.	**CFUW1**	Nodule 1	This study	Milwaukee (WI)	*C*. *fasciculata*
2.	CFUW2	Nodule 4	This study	Milwaukee (WI)	*C*. *fasciculata*
3.	CFUW3	Nodule ZZ1	This study	Milwaukee (WI)	*C*. *fasciculata*
4.	CFUW4*	MSOY	This study	Madison (WI)	*C*. *fasciculata*
5.	CFUSD1*	ASDSOY	This study	Aurora (SD)	*C*. *fasciculata*
6.	CFUSD2*	SDSOY	This study	Sioux Falls (SD)	*C*. *fasciculata*
7.	CFUMN1*	RMN	This study	Rosemount (MN)	*C*. *fasciculata*
8.	CFUMN2*	HUMN	This study	St. Paul (MN)	*C*. *fasciculata*
9.	CFUKY1*	HCOKY	This study	Henderson County (KY)	*C*. *fasciculata*
10.	**USDA3010**	USDA794		Maryland (MD)	*C*. *fasciculata*
11.	USDA3014	USDA797		Not known	*C*. *fasciculata*
12.	USDA3572	USDA798		Tallulah (LA)	*C*. *fasciculata*
13.	USDA3574	USDA800		Percival (IA)	*C*. *fasciculata*
14.	JHI2701*	Cnict		Amazonas, Brazil, Naisbitt et al. (30)	*C*. *nictitans*
15.	JHI2711*	Cfasc		Amazonas, Brazil, Naisbitt et al. (30)	*C*. *fasciculata*
16.	**Cens1A**	BR3804A		Bahia, Brazil, Naisbitt et al., Franco and De Faria (31)	*Chamaecrista ensiformis*
17.	**DW10.1**			Bahia, Brazil, Fonseca et al. (32)	*Dimorphandra wilsonii*
18.	**DW12.5**			Bahia, Brazil, Fonseca et al. (32)	*Dimorphandra wilsonii*

^*a*^ All strains are from the USA unless otherwise noted.

^*b*^ "*” indicates host trap; strains in bold are genome sequenced in this study.

### Amplification and sequencing of genes and phylogenetic analyses

Amplifications of various genes were performed by PCR using genomic DNA as a template; the gene-specific primers and procedures are listed in Table S1. The amplified gene (*rrs*, *recA*, *dnaK*, *nodA*, and *nifH*) products and the amplified 16S-23S ribosomal RNA internal transcribed spacer (ITS) region were purified using the QIAquick PCR purification kit and processed for Sanger sequencing to determine the sequences.

For phylogenetic analyses, various gene sequences of type strains and closely related strains isolated from *Chamaecrista* spp. were retrieved either from genomes or from NCBI-deposited nucleotides and downloaded in FASTA format. The built-in ClustalW (33) tool in Molecular Evolutionary Genetics Analysis (MEGA) software version 7 (34) was used for multiple sequence alignments. Phylogenetic analysis was carried out using the individual and concatenated gene sequences; the phylogenies were reconstructed based on the general time-reversible model using the maximum likelihood method (35) in MEGA. To add confidence values to the branches, bootstrapping of the data set was performed 1,000 times, and the tree branch lengths were measured according to the number of nucleotide substitutions per site.

### Genome sequencing and comparative analysis

Whole-genome-based similarity metrics such as average nucleotide identity (ANI) and digital DNA-DNA hybridization (dDDH) improve taxonomic analysis and assist in defining boundaries for new bacterial species (36, 37). Therefore, draft genome sequences of nine selected strains were obtained through MicrobesNG (https://microbesng.com) using the Illumina HiSeq 2500 sequencing platform (Table 1; Tables S2 and S3). These included four *C. fasciculata* symbionts from the United States (CFUW1, CFUSD1, CFUMN1, and USDA3010) and five from Brazil, three of which were isolated from *Chamaecrista* nodules (JHI2701/Cnict, JHI2711/Cfasc, and Cens1A/BR3804A), while DW10.1 and DW12.5 were isolated from *Dimorphandra wilsonii* nodules (32). Briefly, draft genomes were sequenced using 2 × 250 bp paired-end reads (30× coverage). The raw next-generation sequencing data in Fastq format were subjected to cleaning and removal of adapter sequences. The raw reads were trimmed using Trimmomatic version 0.3 (38). The taxonomic distribution and closest available reference genomes were identified using Kraken 2 (39), and the reads were mapped to the closest *Bradyrhizobium* genome using BWA-MEM (40). The *de novo* assembly of the reads was done using SPAdes (41, 42). Assemblies generated by SPAdes version 3.7 were downloaded in FASTA format, and Quality Assessment Tool for Genome Assemblies (QUAST) version 5.0 by Center for Algorithmic Biotechnology was used for evaluating the genome assemblies (43). To check the completeness and confirm the absence of contamination, the genome assemblies were analyzed through CheckM2 (44) within Galaxy version 1.1. For a circular view, the genomes were further analyzed with CGView Server (45). Annotation was performed using Prokka version 1.11, a pipeline for the rapid annotation of prokaryotic genomes (46). Genes were predicted from the SPAdes-assembled contigs using Prodigal:2.6 (PROkaryotic DYnamic programming Gene-finding ALgorithm) (47).

For each genome sequenced in the present study, the ANI values for pairwise comparisons were computed using FastANI (48) with Galaxy Version 1.3; and BLAST+ (ANIb) and MUMmer (ANIm) were computed through JSpeciesWS, an online service where genomes from the present study were uploaded and compared with genomes of type/reference strains available in the database (49). The ANIm square matrix was used to create a pheatmap (Pretty Heatmaps) in RStudio R 4.4.2 with Bioconductor 3.20 (Galaxy version 24.2+bioc) for visualization, with Euclidean as the default distance method and complete linkage method for clustering strains by similarity (both for rows and columns). ANI heatmap was customized with colors to highlight the standard species boundary of 95%–96% with grouping of strains by their genetic similarity. Similarly, the digital DNA-DNA hybridization similarity values and differences in genomic G+C content were calculated using GBDP2_BLASTPLUS in Genome-to-Genome Distance Calculator 2.1 (https://ggdc.dsmz.de/ggdc.php) (50, 51). Type (Strain) Genome Server (TYGS) (52) and OrthoFinder (53–55) were used to construct a genome-based taxonomy and phylogeny of strains from this study in combination with type/reference *Bradyrhizobium* strains. For annotation of plant growth-promoting traits in bacteria, the PGPT-Pred module of PLaBAse was used with blastp+hmmer as the default program (56). EggNogg-Mapper (57) was used for better prediction of nodulation genes in *Bradyrhizobium* strains.

## RESULTS

### Isolation of symbionts from nodules of *Chamaecrista fasciculata*

**Fig 2:**
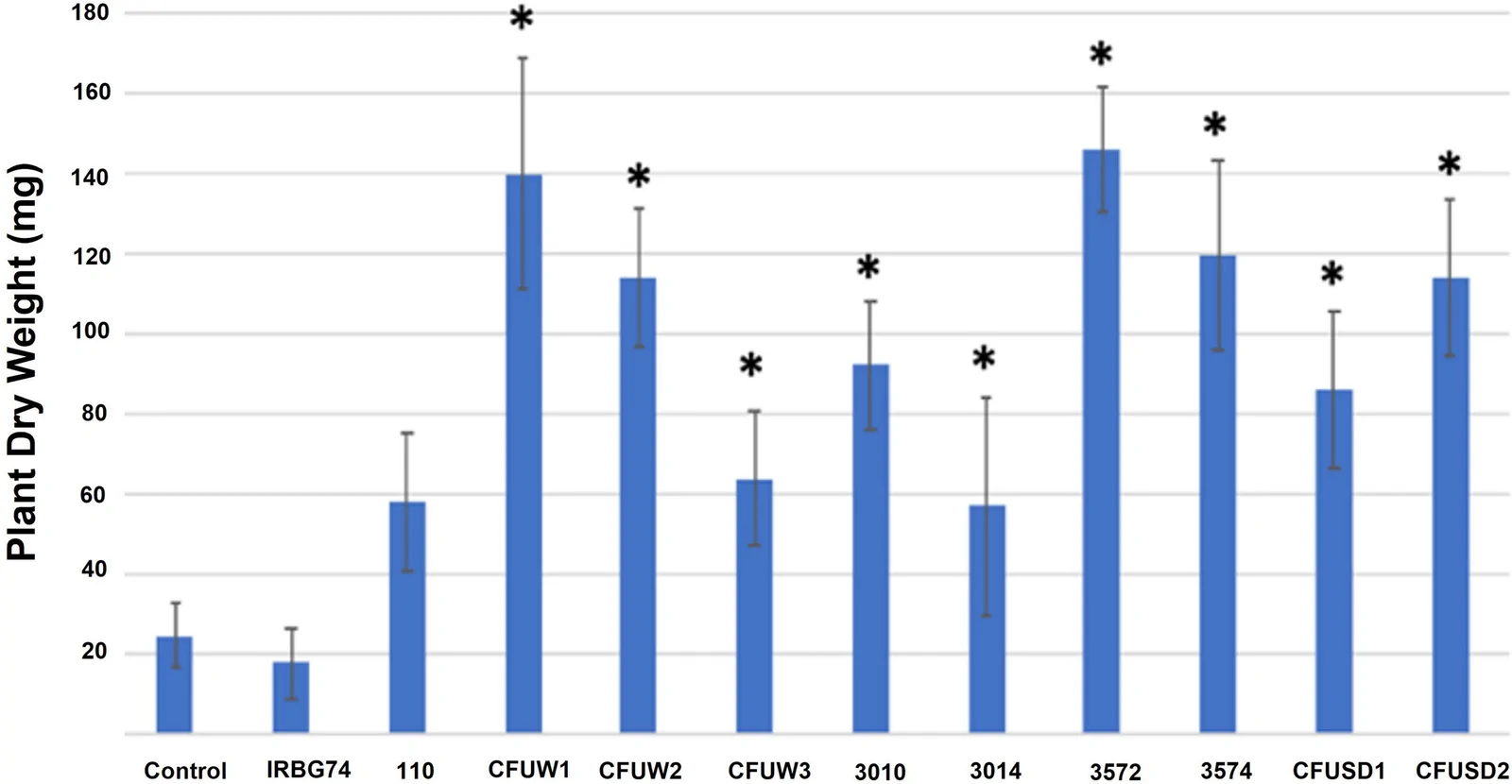
Effect of rhizobial isolates on the growth of *C. fasciculata*. The isolates were inoculated onto seedlings in axenic conditions. Plants were harvested 30 dai, and dry weight was determined. At least five plants were used for each isolate. The results are expressed as mean ± standard deviation. * Significantly different from the uninoculated controls (*P* < 0.05), as determined by *t*-test.

*Chamaecrista fasciculata* plants growing in unmanaged soils at the UW Milwaukee campus (Fig. 1) were found to be nodulated. In addition, *C. fasciculata* nodulated with all soils tested, except for Georgia red clay, Madison, Wisconsin cornfield soil, Purdue, Indiana cornfield soil, and Morris, Minnesota cornfield soil. The isolates from all the root nodules had similar morphology and growth rates on YM plates. Nine isolates representing all the sites in the United States from which nodules were obtained were selected for further analysis. To these were added four strains obtained from the USDA rhizobial germplasm collection that were isolated from root nodules of *C. fasciculata,* plus three *Chamaecrista* strains derived/recovered from those used by Naisbitt et al. (30), which were originally isolated from nodules of *C. fasciculata*, *C. nictitans,* and *C. ensiformis* in Brazil (31). The strains examined in the present study and their origins are listed in Table 1. All the US-derived strains tested (nine in total) were capable of nodulating *C. fasciculata* and promoting its growth (Fig. 2).

### Core phylogeny of *Bradyrhizobium* strains nodulating *Chamaecrista fasciculata* and other *Chamaecrista* species

**Fig 3:**
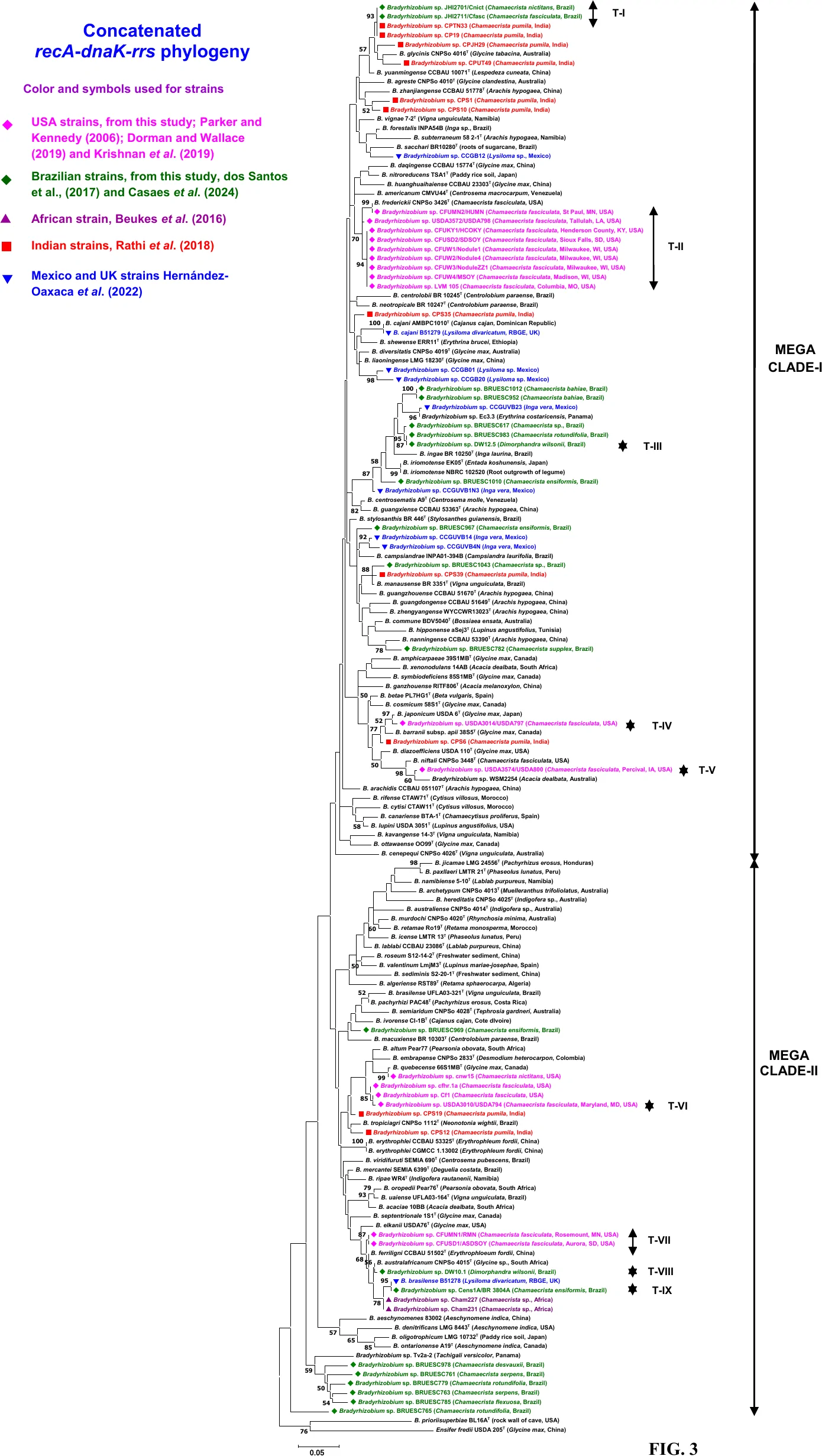
Maximum likelihood phylogeny constructed using *recA-dnaK-rrs* concatenated gene sequences of *Bradyrhizobium* strains isolated from root nodules of *Chamaecrista* sp. in the United States and Brazil, which are compared with previously reported *Bradyrhizobium* strains isolated from various species of *Chamaecrista* in the Old and New World, along with formally and informally described type strains of *Bradyrhizobium*. Bootstrap values greater than 50% calculated for 1,000 replications are indicated at internodes. The scale bar indicates 5% nucleotide substitutions per site.

Based on 16S rRNA sequences, 13 strains isolated from *C. fasciculata* growing in soils from Midwestern and eastern United States (CFUW1, CFUW2, CFUW3, CFUW4, CFUSD1, CFUSD2, CFUMN1, CFUMN2, CFUKY1, USDA3010, USDA3014, USDA3572, and USDA3574) and three Brazilian strains (JHI2701, JHI2711, and Cens1A) were identified as belonging to *Bradyrhizobium* (Table S2); however, the 16S rRNA phylogeny showed poor resolution as the strains clustered with type strains of *Bradyrhizobium* in both Mega Clades-I and II (not shown). For robust phylogenetic analysis, the 16 strains were further analyzed based on sequences of their ITS region (Fig. S1) and two protein-coding housekeeping genes (*recA* and *dnaK*). On concatenation of the *recA + dnaK + rrs* sequences in an MLSA, 13 strains (12 from *Chamaecrista* plus DW12.5) clustered in Mega Clade-I and 5 (4 from *Chamaecrista* plus DW10.1) clustered in Mega Clade-II; these 18 strains were dispersed within five of the nine distinct genotypes labeled T-I to T-IX in Fig. 3. Of the 13 strains in Mega Clade-I, two Brazilian strains (JHI2701 and JHI2711) clustered with Indian *Chamaecrista pumila* symbionts (16) without any close type strains in T-I. Six strains (CFUW1, CFUW2, CFUW3, CFUW4, CFUSD2, and CFUKY1) clustered in genotype T-II along with strain LVM 105, which was isolated from root nodules of *C. fasciculata* in Columbia, Missouri (MO), by Krishnan et al. (23). Strain USDA3572 slightly diverged from this tight cluster within T-II, while strain CFUMN2 shared 100% similarity with *B. frederickii* CNPSo 3426^T^ isolated from *C. fasciculata* growing in MO (26). Therefore, the presence of the type strain within this cluster suggests that members of T-II most likely belong to *B. frederickii*. Of the remaining Mega Clade-I strains, genotype T-III contained no US strains but only strains from Brazilian *Chamaecrista* species (17), plus strain DW12.5 from the Brazilian endemic Caesalpinioid tree *D. wilsonii* (32). However, strain USDA3014 clustered with the soybean symbiont *Bradyrhizobium japonicum* USDA6^T^ in T-IV, while T-V comprised USDA3574, together with the Australian *Acacia dealbata* strain WSM2254, and was related to *B. niftali* CNPSo 3448^T^, which was also isolated from *C. fasciculata* growing in MO (25).

Of the three strains from the present study (USDA3010, CFUSD1, and CFUMN1) that were dispersed within *Bradyrhizobium* Mega Clade-II, these were joined by three previously isolated strains from *Chamaecrista* species in the United States (Fig. 3). Genotype T-VI comprised strain USDA3010, which was divergent from type strains *Bradyrhizobium quebecense* 66S1MB^T^ and *Bradyrhizobium embrapense* CNPSo 2833^T^, but clustered with *Bradyrhizobium* sp. Cf1, isolated from nodules of *C. fasciculata* in Connecticut by Parker and Kennedy (21). Strains cfhr.1a and cnw15, isolated, respectively, from *C. fasciculata* and *C. nictitans* in Mississippi (24), were relatively close to the type strains *B. quebecense* and *B. embrapense*. Meanwhile, strains CFUSD1 and CFUMN1 were identical to each other and clustered close to the Chinese *Erythrophleum fordii* symbiont, *B. ferriligni* CCBAU 51502^T^ in T-VII, but were distant from the South African *Chamaecrista* strains (Cham227 and Cham231). Finally, T-VIII and T-IX were closely related to each other, with the former comprising another Brazilian strain (DW10.1) from *D. wilsonii* (32), while T-IX comprised strain Cens1A from the *Chamaecrista* tree species *C. ensiformis,* plus strain B51278 from *Lysiloma divaricatum*, RBGE, UK (58). Interestingly, however, Cens1A was unrelated to *Bradyrhizobium* strains isolated from other Brazilian endemic species, including its host *C. ensiformis* ([12, 17] and this study). Moreover, none of the US strains clustered with symbionts of *Chamaecrista* from its main center of radiation in Brazil.

### Phylogenetic analysis of symbiosis-essential genes from *Bradyrhizobium* strains nodulating *Chamaecrista fasciculata* and other *Chamaecrista* species

**Fig 4:**
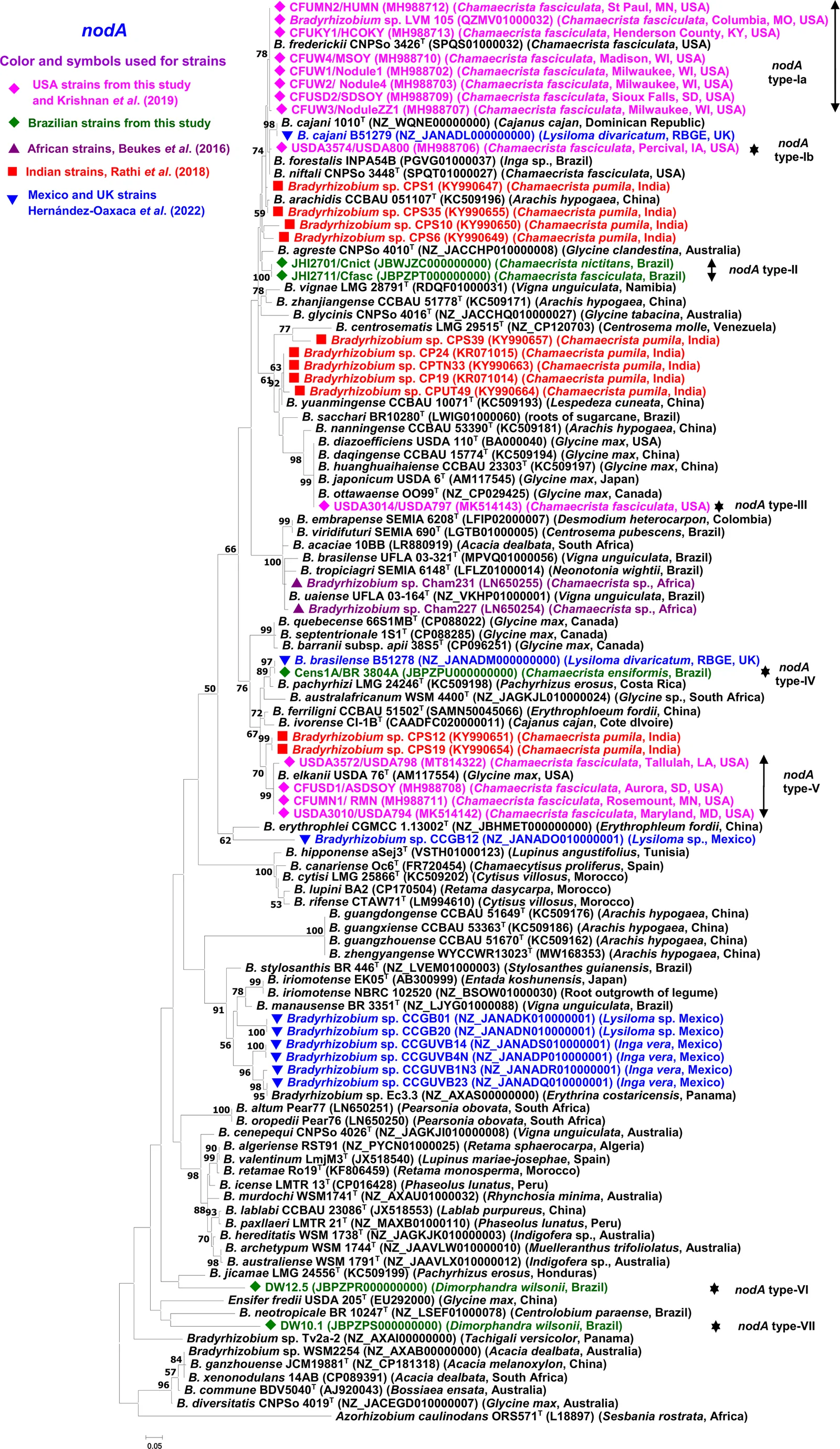
Maximum likelihood phylogeny constructed using *nodA* gene sequences of *Bradyrhizobium* strains isolated from root nodules of *Chamaecrista* sp. in the United States and Brazil, compared with previously reported *Bradyrhizobium* strains isolated from various species of *Chamaecrista* in the Old and New World, along with formally and informally described type strains of *Bradyrhizobium*. Bootstrap values greater than 50% calculated for 1,000 replications are indicated at internodes. The scale bar indicates 5% nucleotide substitutions per site. Accession numbers from GenBank are in parentheses.

Two symbiotic genes (*nodA* and *nifH*) were analyzed from *Bradyrhizobium* strains nodulating *C. fasciculata* and other *Chamaecrista* species; these were compared with symbionts of various other nodulated members of the Caesalpinioideae. The *nodA* phylogeny showed that the *Chamaecrista* strains belonged to seven distinct genotypes (Fig. 4). The *nodA* type-Ia included seven US *C. fasciculata* strains (CFUW1, CFUW2, CFUW3, CFUW4, CFUSD2, CFUMN2, and CFUKY1), clustering with the *B. frederickii* type strain (CNPSo 3426^T^) and LVM 105, both also isolated from *C. fasciculata* in the United States. Meanwhile, strain USDA3574, representing *nodA* type-Ib, clustered with *B. niftali* CNPSo 3448^T^, also isolated from *C. fasciculata* in the United States (25), as well as with *“B. forestalis”* INPA54B^T^, which was isolated from an *Inga* species in Brazil (59). The Brazilian strains JHI2701 and JHI2711, with *nodA* gene sequences, were divergent from existing type strains and formed a distinct *nodA* type-II. Strain USDA3014, representing *nodA* type-III, grouped with three type strains originally isolated from soybean (*Bradyrhizobium daqingense* CCBAU 15774^T^, *Bradyrhizobium huanghuaihaiense* CCBAU 23303^T^, and *B. japonicum* USDA 6^T^). Meanwhile, in Mega Clade-II, the Brazilian symbiont Cens1A, isolated from the tree species *C. ensiformis,* clustered in *nodA* type-IV with strain B51278, isolated from *L. divaricatum* in RBGE, UK (58). In addition, in Mega Clade-II, the *nodA* type-V included three *C. fasciculata* strains (CFUSD1, CFUMN1, and USDA3010), and these showed similarity with the soybean symbiont, *B. elkanii* USDA 76^T^, as did another *C. fasciculata* strain, USDA3572, which contrasts with its position close to *B. frederickii* in Mega Clade-I based on its core gene phylogeny. Finally, the *nodA* types-VI and VII contained the *D. wilsonii* symbionts, DW12.5 and DW10.1, respectively, but no other strains (Fig. 4).

**Fig 5:**
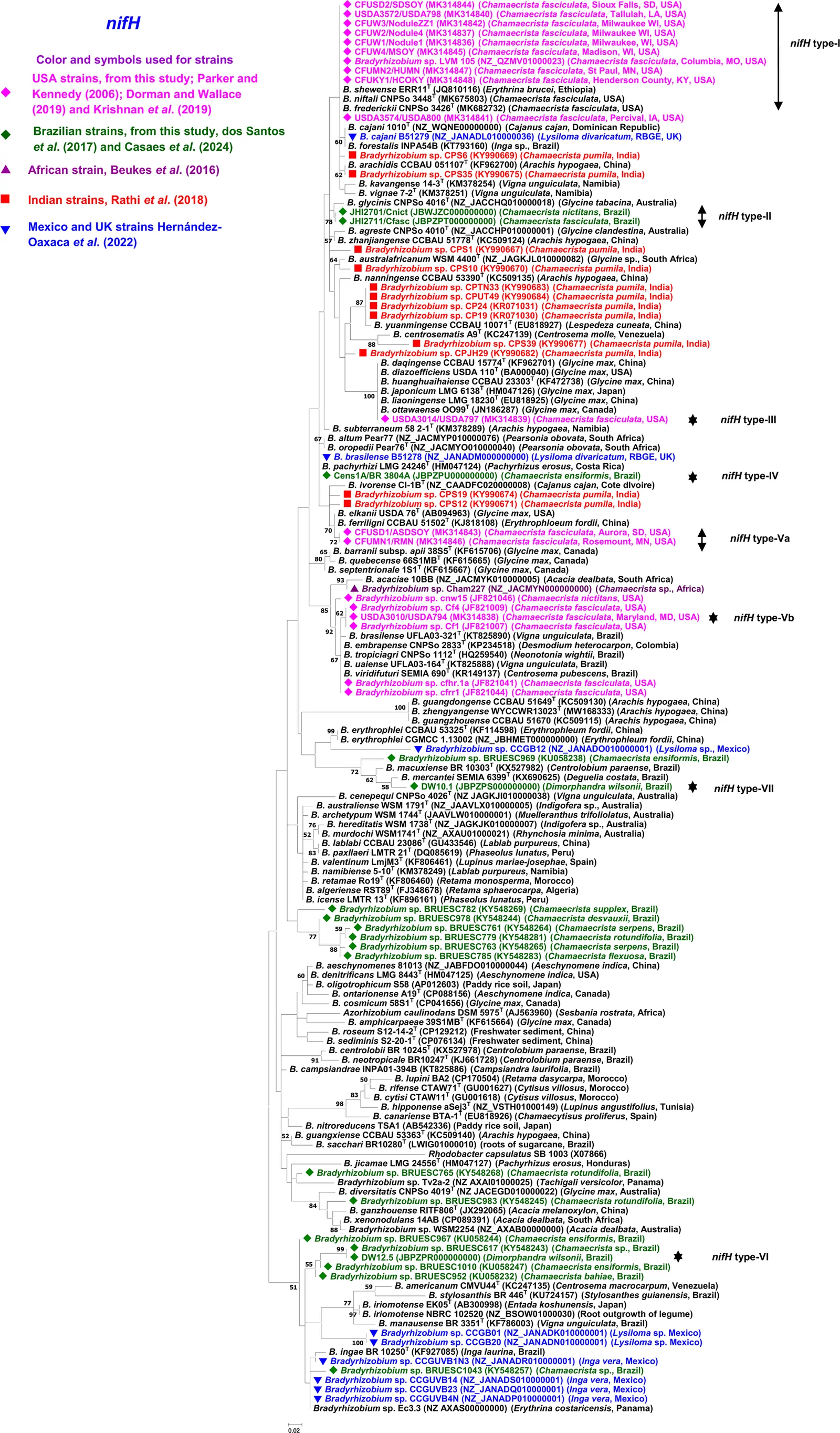
Maximum likelihood phylogeny constructed using *nifH* gene sequences of *Bradyrhizobium* strains isolated from root nodules of *Chamaecrista* sp. in the United States and Brazil compared with previously reported *Bradyrhizobium* strains isolated from various species of *Chamaecrista* in the Old and New World, along with formally and informally described type strains of *Bradyrhizobium*. Bootstrap values greater than 50% calculated for 1,000 replications are indicated at internodes. The scale bar indicates 2% nucleotide substitutions per site. Accession numbers from GenBank are in parentheses.

The *nifH* gene phylogeny (Fig. 5) was more or less congruent with the *nodA* phylogeny, e.g., the 10 *C. fasciculata* strains of Mega Clade-I (CFUW1, CFUW2, CFUW3, CFUW4, CFUSD2, CFUMN2, CFUKY1, USDA3572, USDA3574, and LVM 105) representing *nifH* type-I clustered with the US *C. fasciculata* symbionts *B. frederickii* and *B. niftali*, but also with *B. shewense* ERR11^T^ isolated from *Erythrina brucei* in Ethiopia (60). The *nifH* sequences of the Brazilian *Chamaecrista* strains JHI2701 and JHI2711 (comprising *nifH* genotype-II) shared close similarities with the *Glycine* spp. symbionts *B. glycinis* CNPSo 4016^T^ (61) and *B. zhanjiangense* CCBAU 51778^T^, while strain USDA3014 representing *nifH* genotype-III clustered in the *B. japonicum* clade along with other soybean symbionts, such as *B. daqingense*, *B. huanghuaihaiense*, *B. liaoningense* LMG 18230^T^, and *B. ottawaense* OO99^T^. Meanwhile, two *C. fasciculata* strains in Mega Clade-II (CFUSD1 and CFUMN1) formed a cluster (*nifH* genotype-Va) close to *B. elkanii* and *B. ferriligni*, but strain USDA3010 (*nifH* genotype-Vb), similar to its core gene phylogeny, diverged from these in the *nifH* phylogeny and clustered with previously reported microsymbionts of *C. fasciculata* from the USA, i.e.*, Bradyrhizobium* sp. Cf1 and Cf4 (24, 28). The combined *nifH* genotype-Va and -Vb, containing three *C. fasciculata* strains from this study plus other US strains, was related to but diversified from the type strains of *B. embrapense* CNPSo 2833^T^, “*B. brasilense”* UFLA03-321^T^, *Bradyrhizobium tropiciagri* CNPSo 1112^T^, *Bradyrhizobium uaiense* UFLA03-164^T^, and *Bradyrhizobium viridifuruti* SEMIA 690^T^ (Fig. 5). The Brazilian *C. ensiformis* strain Cens1A clustered in *nifH* type-IV with *Bradyrhizobium pachyrhizi* LMG 24246^T^ (from *Pachyrhizus erosus*, Costa Rica) and among the two Brazilian *D. wilsonii* symbionts, strain DW12.5 (comprising *nifH* genotype-VI), shared similarity with BRUESC617 (isolated from *Chamaecrista* sp., Brazil), and DW10.1 (comprising *nifH* genotype-VII) clustered close to other Brazilian strains, such as *Bradyrhizobium mercantei* SEMIA 6399^T^ (from *Deguelia costata*), *Bradyrhizobium macuxiense* BR 10303^T^ (from *Centrolobium paraense*), and BRUESC969 (from *C. ensiformis* var. *plurifoliolata*).

### Comparative genomic analysis of selected *Bradyrhizobium* strains

**Fig 6:**
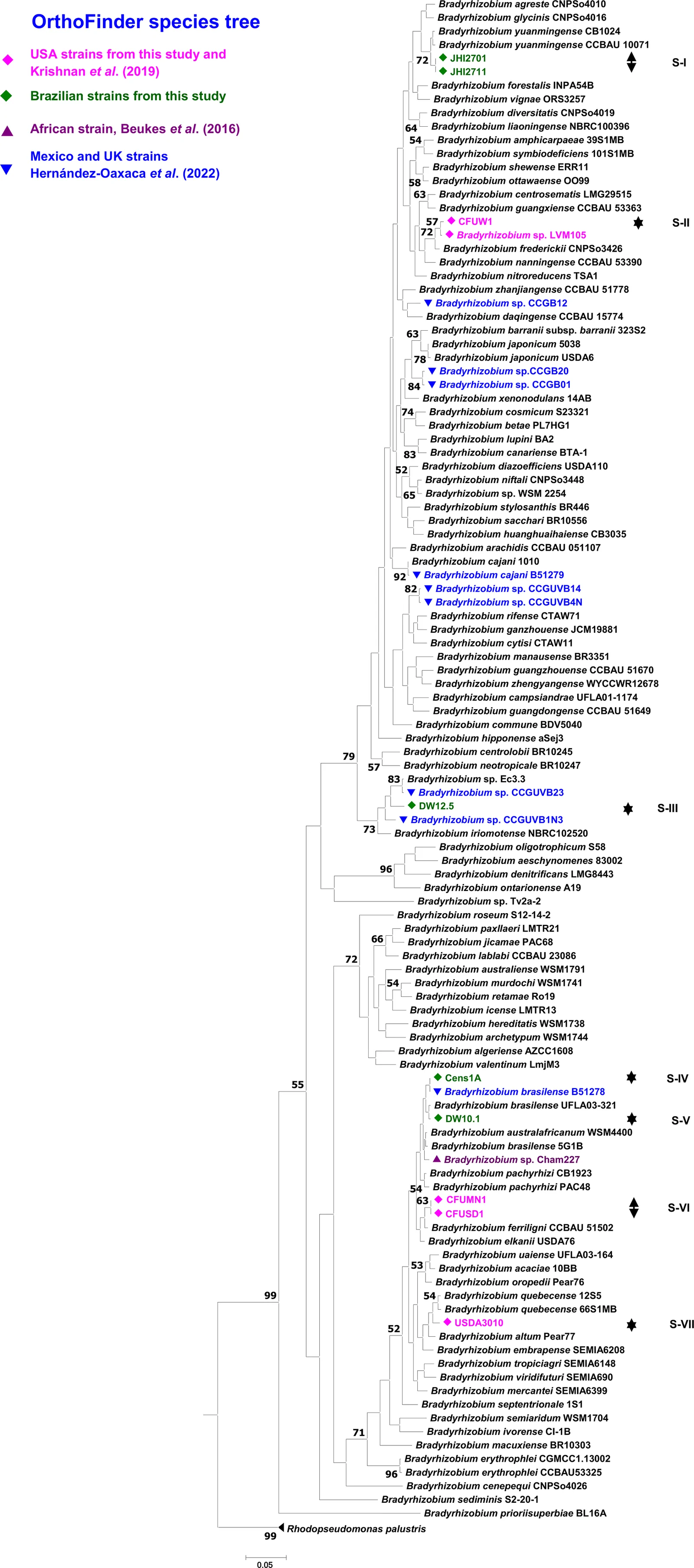
OrthoFinder species phylogeny: amino acid sequences of 120 strains were used as input in OrthoFinder version 2.5.5 with the DIAMOND program for sequence similarity searches to construct a species tree. The species tree was inferred by the STAG (species tree inference from all genes) algorithm and rooted by the STRIDE (species tree root inference from gene duplication events) algorithm. OrthoFinder assigned 912,782 genes (98.3% of total) to 23,879 orthogroups. Fifty percent of all genes were in orthogroups with 118 or more genes, G50 (all genes). The number of orthogroups with all species present was 1,369. *Rhodopseudomonas palustris* strains were used as an outgroup. The scale bar represents 5% substitutions per site.

Sequence coverage, number of reads, etc., of the genomes of the seven *Chamaecrista Bradyrhizobium* strains selected for sequencing are given in Table S3, while complete assembly statistics calculated using QUAST are presented in Table S4. An assessment of genome assembly quality, completeness, and contamination done through checkm2 (Galaxy Version 1.1.0+galaxy0) is presented in Table S5. The overall genome-relatedness indices calculated for the seven *Chamaecrista* strains plus two *Dimorphandra* strains (DW10.1 and DW12.5) (Fig. S2A and B), in comparison with publicly available *Bradyrhizobium* genomes, including formally and informally named type strains (Table S6), are presented in Fig. S3A and B and Table S7. Overall comparative genomics statistics in inferring orthologous genes among 120 strains via OrthoFinder are presented in Table S8 with details of the number of genes in the orthogroups, the number of orthogroups with all species present, together with per-species statistics such as the number of unique genes and species-specific orthogroups. In the species phylogeny using the STAG (species tree inference from all genes) algorithm, two of the Brazilian strains, JHI2701 and JHI2711, appear to be clones, and they cluster with the Chinese *Lespedeza* symbiont *B. yuanmingense* in orthogroup S-I (Fig. 6). Meanwhile, the *C. fasciculata* strains CFUW1 and LVM 105 comprised genomotype S-II, and both were closely related to *B. frederickii,* also originally isolated from *C. fasciculata* in the United States (26), and as also indicated in the MLSA (Fig. 3), they most probably belong to this species. The orthogroup S-IV comprised the Brazilian strain Cens1A/BR8034a isolated from the fixation thread (FT)-type nodule-forming tree species *C. ensiformis*; it clustered with strains of “*B. brasilense”* isolated from nodules of cowpea trap plants in Brazil or from *L. divaricatum* nodules in the UK RBGE (58, 62). The genomes of the *C. fasciculata* strains CFUSD1 and CFUMN1 comprised orthogroup S-VI, and as observed in the MLSA (Fig. 3), shared similarities with *B. elkanii* and *B. ferriligni* of Mega Clade-II. As per the generally accepted species designation threshold values for ANI (> 95% same species) and dDDH (> 70% same species), the single US *C. fasciculata* strain USDA3010, comprising orthogroup S-VII, is a potential new species of *Bradyrhizobium*. The remaining orthogroups containing genomes from the present study were S-III and S-V; these contained no *Chamaecrista* symbionts but did contain symbionts of related trees in the Brazilian endemic non-Mimosoid Caesalpinioideae, namely DW10.1 (S-V) and DW12.5 (S-III) from *D. wilsonii*. Moreover, as with the symbiotic gene loci (*nodA*; Fig. 4 and *nifH*; Fig. 5), the few other available genomes of microsymbionts of other non-mimosoid Caesalpinioideae species, namely *Bradyrhizobium camsiandrae* (from *Campsiandra laurifolia*), *Bradyrhizobium erythrophlei* and *Bradyrhizobium ferriligni* (both from *Erythrophleum fordii*), and *Bradyrhizobium* sp. TV2a-2 (from *Tachigali versicolor*), all of whose hosts form nodules with their bacteroids housed in FTs (11), were scattered across the *Bradyrhizobium* phylogenome and were not affiliated with any particular orthogroup or species (Fig. 6).

**Fig 7:**
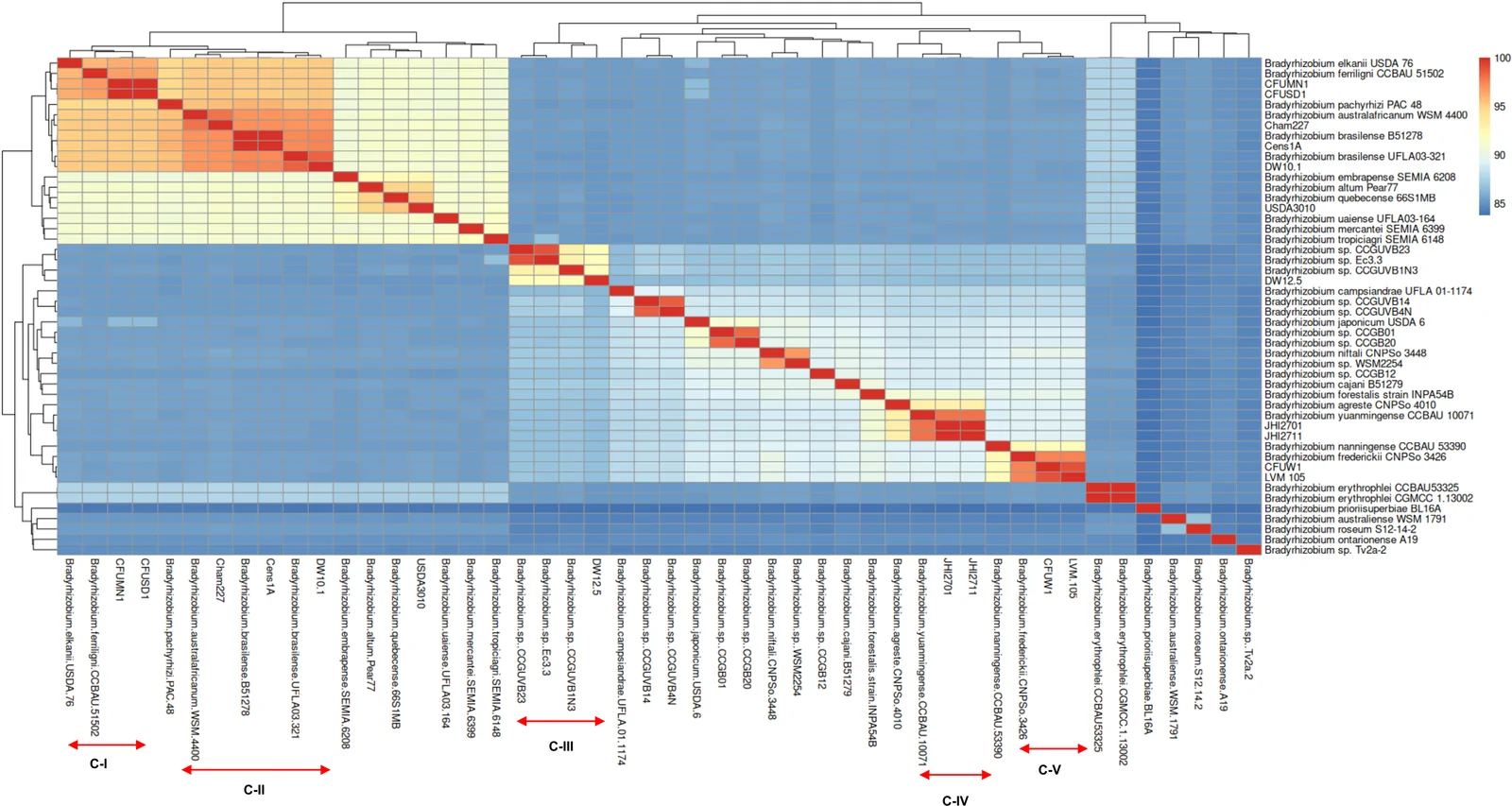
Pheatmap (pretty heatmaps) was created with average nucleotide identity (ANIm) of *Bradyrhizobium* strains from the present study with type/reference strains based on MUMmer results. ANIm values (percentage identity) in a square matrix format were imported into RStudio R 4.4.2 with Bioconductor 3.20 (Galaxy Version 24.2+bioc) for visualization, with Euclidean as the default distance method and complete linkage method for clustering strains by similarity (both for rows and columns). ANI Heatmap was customized with colors to highlight the standard species boundary of 95%–96% with grouping of strains by their genetic similarity.

In the TYGS-whole genome-based phylogeny, including strains from this study along with previously reported *Bradyrhizobium* strains isolated from species of *Chamaecrista*, non-mimosoid and mimosoid members of the Caesalpinioideae, as well as *Bradyrhizobium* type/reference strains, the grouping of strains (Fig. S4) is sufficiently congruent with the species phylogeny based on orthogroups to infer their evolutionary relationships (Fig. 6). Phylogenomic results were further supported using ANIm (alignment of complete sequences of two genomes using MUMmer’s algorithm [MUMs—Maximal Unique Matches] computed with JSpeciesWS tool), a similarity matrix-based clustering of strains in pheatmap with complete linkage method (Fig. 7). The first major cluster comprised strains CFUSD1 and CFUMN1 from Mega Clade-II of *Bradyrhizobium* sharing maximum similarities with *B. ferriligni* (96.7%) and *B. elkanii* (96%). The second cluster comprised Cens1A, which was identical to B51278 as observed in individual core and *sym* genes, as well as genome-based phylogenies. It shared 97% similarity with DW10.1 from *D. wilsonii* (32), the South African strain Cham227 (20), *B. australafricanum* (63), and “*B. brasilense”* (62). In Cluster-III, USDA3010 shared approximately 95% similarities with *B. quebecense* 66S1MB^T^ and *B. altum* Pear77, but as per FastANI and ANIb, similarities with these strains were less than 95%, and the dDDH (Table S7) values also supported USDA3010 as a potential new species of *Bradyrhizobium*. In Cluster-IV, DW12.5 from *D. wilsonii* shared only 92% similarity with symbionts of *Inga vera* (CCGUVB1N3 and CCGUVB23) from Mexico (58) as well as with *Bradyrhizobium* sp. Ec3.3 (isolated from the Papilionoid host *Erythrina costaricensis* in Panama). Meanwhile, strains JHI2701 and JHI2711 clustered alongside *B. yuanmingense* CCBAU 10071^T^ with 98% similarity. Similarly, strains CFUW1 and LVM 105 clustered with *B. frederickii,* sharing 97% similarity with the type strain CNPSo 3426^T^.

Nodulation (*nod*, *noe,* and *nol*) genes were predicted using EggNogg-Mapper and the PGPT-Pred tool of PLaBAse to compare the PGPT gene frequencies in strains from this study with some closely related and type/reference strains of *Bradyrhizobium* (Table S9). Among the core genes related to the synthesis of nod factors (NFs), variation was observed for *nodE*, which was exclusively present in the novel strain DW12.5 among all the strains compared. The *nodE* gene encodes beta-ketoacyl synthase that catalyzes the condensation reactions and determines the specific length and structure (saturation) of the fatty acyl chain attached to the N-acetylglucosamine backbone of the NF molecule. Such modification is a major determinant of host specificity; in some species, NodE and NodF (an acyl carrier protein) function together to modify the NF fatty acid tail (64). Among the specific genes related to modifications of the Nod signal, *nodL* encodes a host-specific nodulation protein that catalyzes the addition of an acetyl group at the terminal non-reducing end of the lipo-chitooligosaccharide backbone, which was absent in *B. frederickii* strains (CFUW1, LVM105, and CNPSo 3426^T^) and two *B. yuanmingense*-like Brazilian strains (JHI2701 and JHI2711) but was present in *B. yuanmingense* CCBAU 10071^T^. The *nodX* gene encodes an acetyltransferase that transfers a specific acetyl group at the C-6 position of NF, which is recognized by specific hosts, and was absent in all strains from this study and related type strains, while *noeC* encodes a protein involved in the arabinosylation of the NF and was absent in the *C. fasciculata* strains CFUW1, LVM105, CNPSo 3426^T^, and USDA3010. Another gene for host specificity of nodulation (*hsn*) is *nolO*, which encodes a protein (NolO) responsible for adding a carbamoyl group to the non-reducing end of NF; this was missing in only DW12.5 and USDA3010, whereas *nolL* (which encodes O-acetyltransferase) and *nolR* (which encodes a transcriptional repressor) genes were absent in all of them. Of the four genes (*nod*IJTO) whose products are responsible for the secretion and transport of Nod signals out of the bacterial cell, *nod*IJ were present in all, *nodO*, encoding a calcium-binding pore-forming protein, was absent in all strains of this study, while *nodT,* coding for the outer membrane protein, was present only in *B. frederickii* strains (CFUW1, LVM105, CNPSo 3426^T^, and USDA3010) and the *T. versicolor* symbiont Tv2a-2. These variations in the presence or absence of various nodulation genes and even the commonalities among the closely related *Bradyrhizobium* strains indicate their adaptation for symbiotic abilities, particularly regarding host specificity.

## DISCUSSION

### *Chamaecrista fasciculata*-nodulating symbionts in US soils are *Bradyrhizobium*

Previous studies have characterized the symbionts of the nodulating caesalpinioid legume *C. fasciculata* growing in soils of the United States (23–26). To further extend our understanding of *C. fasciculata* root nodule symbionts in the United States, we demonstrate that *C. fasciculata* is nodulated in non-agricultural soils of WI, which is in its native range. In addition, functional nodules were formed when soils from different parts of the United States were used as inocula for *C. fasciculata* as a host trap. Rhizobia from these nodules were isolated, and selected strains were confirmed for their ability to form symbioses with *C. fasciculata* when it was grown under axenic conditions. All the selected strains formed nodules and enhanced the growth of *C. fasciculata* in N-free conditions, indicating effective symbiotic interactions. There were, however, significant differences in the growth promotion ability of these strains, indicating functional and likely genotypic diversity of the strains (Fig. 2). The *C. fasciculata*-nodulating strains isolated from soils of different states of the United States (Wisconsin, South Dakota, Minnesota, Kentucky, Maryland, Louisiana, and Iowa) were shown to be phylogenetically diverse species forming clades/clusters and lineages in both Mega Clades of the genus *Bradyrhizobium*. Previously reported *Bradyrhizobium* strains from *C. fasciculata* in the United States were mainly in Mega Clade-I, such as *B. frederickii* from Oregon and Missouri (26), *B. niftali* from Missouri (25), and strain LVM 105 also from Missouri (23), but a few strains from Mega Clade-II have also been reported, such as strain Cf1 from Connecticut and strain cfhr.1a from North Carolina (24). Similarly, in the present study, eight of the US *Bradyrhizobium* strains (CFUW1, CFUW2, CFUW3, CFUW4, CFUSD2, CFUMN2, CFUKY1, and USDA3572) represented a major genotype (T-II) from Mega Clade-I that showed close relatedness to *B. frederickii* (26) and to strain LVM 105, indicating that *B. frederickii* and *B. frederickii*-like strains are common symbionts of *C. fasciculata* and are widespread in different states of the United States.

Based on the housekeeping gene MLSA, USDA3574 formed a distinct lineage (T-V) and shared close similarity with *B. niftali*, also a symbiont of *C. fasciculata* (25). Unlike other strains, USDA3014 (T-IV) clustered with *B. japonicum*. Of the three strains positioned within Mega Clade-II, two of them (CFUSD1 and CFUMN1) formed a phylogenetically distinct cluster (T-VII), diversified from *B. ferriligni,* while strain USDA3010 (T-VI) shared similarities with earlier reported *C. fasciculata* strains from the United States (24). Interestingly, the Brazilian strains JHI2701 and JHI2711 (T-I) shared close similarities with *B. yuanmingense* CCBAU 10071^T^ (65) and were identical to the dominant group of “*B. yuanmingense*-type” symbionts reported from root nodules of *C. pumila* (16), *Tephrosia* spp. (66), and *Vigna* spp. (67), growing in alkaline to neutral soils in India. A Brazilian symbiont of *C. ensiformis* from this study, Cens1A (T-IX), shared affinities with a symbiont of *L*. *divaricatum*, B51278. Additionally, one of the Brazilian *Bradyrhizobium* strains from *D. wilsonii*, DW10.1 (T-VIII), was closely related to *B. australafricanum* (*Glycine* sp., South Africa) (63), while another, DW12.5 (T-III), clustered with the Brazilian strains BRUESC983 (from *Chamaecrista rotundifolia*) and BRUESC617 (*Chamaecrista* sp.) (17). Brazil (South America) is the center of radiation for the large genus *Chamaecrista,* and several species from all sections of the genus (for example, *Chamaecrista bahiae, Chamaecrista desvauxii*, *C. ensiformis*, *Chamaecrista flexuosa*, *C. rotundifolia*, *Chamaecrista serpens,* and *Chamaecrista supplex*) have been studied for their root nodule microsymbionts (12, 17). Diverse species of *Bradyrhizobium* have been reported to nodulate species of *Chamaecrista* from different continents like Africa, Asia, South America, and North America (12, 16–18, 20, 23–26). Other non-mimosoid members of the Caesalpinioideae have also been described to be nodulated by species of *Bradyrhizobium* (32, 68–70).

Of the seven *Chamaecrista-Bradyrhizobium* strains (CFUW1, CFUSD1, CFUMN1, USDA3010, JHI2701, JHI2711, and Cens1A) from the United States and Brazil and the two Brazilian *Bradyrhizobium* strains from *D. wilsonii* (DW10.1, DW12.5) analyzed at the genomic level, using multiple comparative genomic tools such as ANI, dDDH, and phylogenies based on whole genomes (Genome BLAST Distance Phylogeny using TYGS) and on orthologous groups, strain USDA3010 is a potential new species diversified from *B. quebecense* 66S1MB^T^ (isolated from *Glycine max*, Canada), *B. altum* Pear77 (isolated from *Pearsonia obovata*, South Africa), and *B. embrapense* SEMIA 6208^T^ (isolated from *Desmodium heterocarpon*, Colombia). Likewise, DW12.5 is a potential new species related to strains (CCGUVB1N3 and CCGUVB23) isolated from *Inga vera* in Mexico (58). The remaining seven strains shared higher percentage ANI and dDDH similarities with type strains of *B. yuanmingense*, *B. australafricanum*, “*B. brasilense,” B. pachyrhizi*, *B. elkanii, B. ferriligni*, but particularly of *B. frederickii* and strain LVM 105, suggesting concordance in the MLSA and genomotaxonomy data.

### Diversity of symbiotic genes of *Bradyrhizobium* strains nodulating *Chamaecrista* in the United States

In general, congruence was observed in core and symbiotic (*nodA* and *nifH*) gene phylogenies. This is common for the genus *Bradyrhizobium,* as it possesses its symbiotic genes on chromosomal symbiotic islands (symbiotic integrative and conjugative elements), which tend to be associated with the conserved chromosomal sites. They are integrated into the chromosome and undergo excision before they can be transferred; hence, they are not so easily subject to horizontal gene transfers (71–73). The *sym* genes of eight *C. fasciculata* strains were similar to those of other symbionts (*B. frederickii*, *B. niftali,* and strain LVM 105) isolated from *C. fasciculata* in the United States, suggesting their co-evolution. This likelihood is reinforced by strain USDA3572 of Mega Clade-I, which was closely related to *B. frederickii* in the core gene phylogeny but grouped with three *C. fasciculata* strains (CFUSD1, CFUMN1, and USDA3010) in Mega Clade-II, along with the soybean symbiont *B. elkanii* USDA 76^T^ in the *nodA* gene phylogeny. Interestingly, regarding soybean, strain USDA3014 of Mega Clade-I had *nodA* and *nifH* genes identical to *B. japonicum* and the other soybean-nodulating strains clustering in this clade, such as *B. daqingense*, *B. huanghuaihaiense*, *B. liaoningense,* and *B. ottawaense*. Similarly, congruence was observed in the *nodA* and *nifH* gene phylogenies of the *B. yuanmingense*-type strains JHI2701 and JHI2711 from Brazil. There are several reports of genetically diverse *Bradyrhizobium* strains possessing common *nod* sequences (73), but based on the *nifH* phylogeny, strains CFUSD1 and CFUMN1 also formed a distinct cluster for this symbiotic locus and shared close similarities with *B. ferriligni* CCBAU 51502^T^ (isolated from root nodules of the caesalpinioid tree *Erythrophleum fordii* in China) and *B. elkanii* USDA 76^T^. Meanwhile, the *nifH* sequence of the potential new species represented by strain USDA3010 grouped with other US *Bradyrhizobium* strains from *C. fasciculata* (24) and shared high similarity with “*B. brasilense,” B. embrapense*, *B. tropiciagri*, *B. uaiense,* and *B. viridifuruti*. The Brazilian strain Cens1A was identical to B51278 and shared affinities with *B. pachyrhizi*. The two *D*. *wilsonii* symbionts DW10.1 and DW12.5 carry distinct nodulation genes; however, at *nifH* loci, DW10.1 shared similarities with *B. mercantei* SEMIA 6399^T^ in contrast to its phylogenomic affinities with *B. australafricanum* and “*B. brasilense.”* Potential new species DW12.5 clustered with Brazilian-*Chamaecrista* strain BRUESC617 (17) in *nifH* phylogeny. Overall, the majority of *C. fasciculata-Bradyrhizobium* strains from the present study were symbiotically distinct from the microsymbionts of *Chamaecrista* sp. reported from India, Africa, and Brazil.

In the present study, “*B. yuanmingense*-type” symbionts were isolated from a very limited sampling in Brazil, which were not reported in the extensive sampling of dos Santos et al. (17) and Casaes et al. (12) on *Chamaecrista* spp. Moreover, some *Bradyrhizobium* strains associated with species of *Chamaecrista* in its center of radiation, Brazil, are divergent and constitute a unique community of microsymbionts (12, 17, 18).

### Fixation threads versus symbiosomes in *Chamaecrista* and other non-mimosoid Caesalpinioideae nodules: do they influence bradyrhizobial symbiont genotype?

In a recent study of Brazilian *Chamaecrista* symbionts isolated from more than 20 species across various biomes in the state of Bahia, a center of radiation for the genus *Chamaecrista*, Casaes et al. (12) examined nodules and (brady)rhizobial symbionts of plants ranging in growth habit from small to medium shrubs growing in nutrient-poor, seasonally dry savannah-type ecosystems to small to large trees growing in the nutrient-rich rainforest. They concluded that cell wall-bound FTs were the standard symbiosome (SYM) type housing bacteroids in nodules on large rainforest trees in the small *Chamaecrista* section Apoucouita, while in the large number of species outside this section, which are mainly savannah-dwelling shrubs, the nodules contained either “standard” membrane-bound symbiosomes or intermediate FT-SYM-type symbiosomes with thin cell walls enclosing their bacteroids. All species, regardless of their growth habit, were nodulated by *Bradyrhizobium* strains, with one exception in the FT-nodule type tree species, *Chamaecrista eitenorum* var. *eitenorum,* which can also be nodulated by *Paraburkholderia* (12).

De Faria et al. (11) had previously noted that all nodulating non-mimosoid Caesalpinioideae genera examined for this trait had FT-type nodules (with the notable exception of the largest and most diverse genus in this group, *Chamaecrista*), and that the FT-trait was linked strongly to losses of nodulation, i.e., very few genera in this group of legumes are nodulated, while their relatives in the tribe Mimosae, which arose from them, are mostly nodulated, but they all have the more evolutionarily advanced and “stable” SYM-type nodules. It has also been noted that the majority of reports of microsymbionts isolated from FT-type nodules identified them as strains of *Bradyrhizobium* (1, 11, 32), suggesting an affinity between bradyrhizobia and the FT type of bacteroid. On the other hand, bradyrhizobia are also very common symbionts of SYM-type nodules, such as those in the tribe Mimosae, e.g., *Inga* and *Lysiloma* (22, 58, 74). Moreover, as already noted for *Chamaecrista* (12, 17), there are no obvious rhizobial genotypes associated with FT or SYM-type nodules in this large genus. However, the aforementioned reports of FT- versus SYM-type bradyrhizobia microsymbionts were based on single amplified loci, and it is likely that whole-genome sequences will be more informative and might reveal loci that are more important for particular bacteroid types. Therefore, the present study sequenced the whole genomes of three bradyrhizobial symbionts of FT-type nodulating tree species, i.e., the *Dimorphandra wilsonii* strains DW10.1 and DW12.5 (32) and the *Chamaecrista ensiformis* strain Cens1A/BR8034a, and compared them with the four other genomes publicly available, i.e., the *Tachigali versicolor* strain Tv2a-2 (69), the *Erythrophleum fordii* strains, *B. erythrophlei* CCBAU 53325^T^ and *B. ferriligni* CCBAU 51502^T^ ([70] and this study), and the *Campsiandra laurifolia*-derived type strain “*B. campsiandrae”* UFLA 01-1174^T^ (75). This genome-based analysis also did not reveal any obvious loci or groups of loci that might be considered “FT-specific,” but this might be expected as only the aforementioned seven strains were included, and these represent only a fraction of FT-nodulating species. Therefore, we propose to conduct a much wider comparative genomic analysis of FT- versus SYM-type nodule bradyrhizobial symbionts from both the genus *Chamaecrista* and the Caesalpinioideae subfamily as a whole, as and when more genomes become available.

### Conclusion

It is clear that *C. fasciculata*, as with most *Chamaecrista* species (12, 16, 17), is mainly nodulated by diverse strains of *Bradyrhizobium*, but it appears to have a particular preference for those in the exclusively (to date) North American species *B. frederickii* (26). Some of these *B. frederickii* strains were genome-sequenced, including strain CFUW1; we propose that the *C. fasciculata-B. frederickii* CFUW1 symbiosis be used as a model system in the Caesalpinioideae subfamily and hence provide an alternative/additional perspective for examining the evolution and functioning of nodulating symbioses to the model symbiotic nodulation systems in its more widely known sister subfamily Papilionoideae, *M. truncatula* and *L. japonicum* (5).

## Data Availability

Genome sequences from the present study have been submitted under BioProject PRJNA1293807. Whole genome shotgun (WGS) project accession numbers are as follows: JBWJZF000000000, JBWJZG000000000, JBWJZE000000000, JBWJZC000000000, JBPZPT000000000, JBPZPU000000000, JBPZPS000000000, JBPZPR000000000, JBWJZD000000000, and JBPZPQ000000000.

## SUPPLEMENTAL MATERIAL

Supplemental figures spectrum.00798-26-s0001.pdf Fig. S1 to S5.

Supplemental tables spectrum.00798-26-s0002.pdf Tables S1 to S9.

ASM does not own the copyrights to Supplemental Material that may be linked to, or accessed through, an article. The authors have granted ASM a non-exclusive, world-wide license to publish the Supplemental Material files. Please contact the corresponding author directly for reuse.
