# Supplementary material for: Comparative genomic analysis of North American Bradyrhizobium symbionts of Chamaecrista fasciculata (Caesalpinioideae) with related bradyrhizobia
Source: Microbiol Spectr. 2026 Aug 19;14(10):e00798-26. doi: 10.1128/spectrum.00798-26 (PMC13689284; doi:10.1128/spectrum.00798-26)
Supplement: Supplementary file 1 [file spectrum.00798-26-s0001.pdf]

Supplementary Materials

Comparative genomic analysis of North American *Bradyrhizobium* symbionts of *Chamaecrista fasciculata* (Caesalpinioideae) with related bradyrhizobia

Nisha Tak[a#], Zachary Zawada[b#], Marta Maluk[c], Chrizelle Beukes[c], Laura Ketelboeter[b], Hukam S. Gehlot[a], Prasad Gyaneshwar[b]*, Euan K. James[c]*

[a]*Department of Botany, Jai Narain Vyas University, Jodhpur - 342001, Rajasthan, India*

[b]*Department of Biological Sciences, University of Wisconsin – Milwaukee, Milwaukee, WI 53211 USA, and* [c]*The James Hutton Institute, Dundee DD2 5DA, UK*

[#]These authors made an equal contribution

**For correspondence. Email* prasadg@uwm.edu, euan.james@hutton.ac.uk

Legends to Supplementary Figures:

FIG. S1 Maximum Likelihood phylogenetic tree using **16S-23S rRNA intergenic spacer (ITS)** gene sequences of ***Bradyrhizobium*** strains isolated from root nodules of ***Chamaecrista* sp.** in USA and Brazil compared with previously reported *Bradyrhizobium* strains isolated from various species of *Chamaecrista* along with type strains of *Bradyrhizobium*. Bootstrap values more than 50% calculated for 1,000 replications are indicated at internodes. The scale bar indicates 10% nucleotide substitution per site. (Abbreviations: *B*., *Bradyrhizobium* and superscript ^{T}, type strain)

FIG. S2 A–B Circular genome view of bacterial chromosome using CGView.js of *Bradyrhizobium* strains from present study: CFUMN1 (9,649,768 bp), CFUSD1 (9,699,723 bp), CFUW1 (8,286,627 bp), USDA3010 (9,181,251 bp), JHI2701 (7,861,681 bp), JHI2711 (7,860,189 bp), Cens1A (8,575,174 bp), DW10.1 (8,844,051 bp), and DW12.5 (9,832,845 bp).

FIG. S3 Heatmap generated using ChiPlot (https://www.chiplot.online/heatmap.html) with default parameters (Euclidean distance and complete linkage method) for Fast-ANI (A) and ANIb (B) percentage similarity matrix of strains from present study with other strains of *Bradyrhizobium*.

FIG. S4 Intergenomic distances were used to infer a balanced minimum evolution **phylogenomic tree** with FastME 2.1.6.1 using Type (Strain) Genome Server (TYGS). The branch lengths are scaled in terms of Genome BLAST Distance Phylogeny (GBDP) distance formula d_5. The numbers above branches are GBDP pseudo-bootstrap support values > 60 % inferred from 100 pseudobootstrap replicates each, with an average branch support of 89.6 %. The tree was rooted at the midpoint.

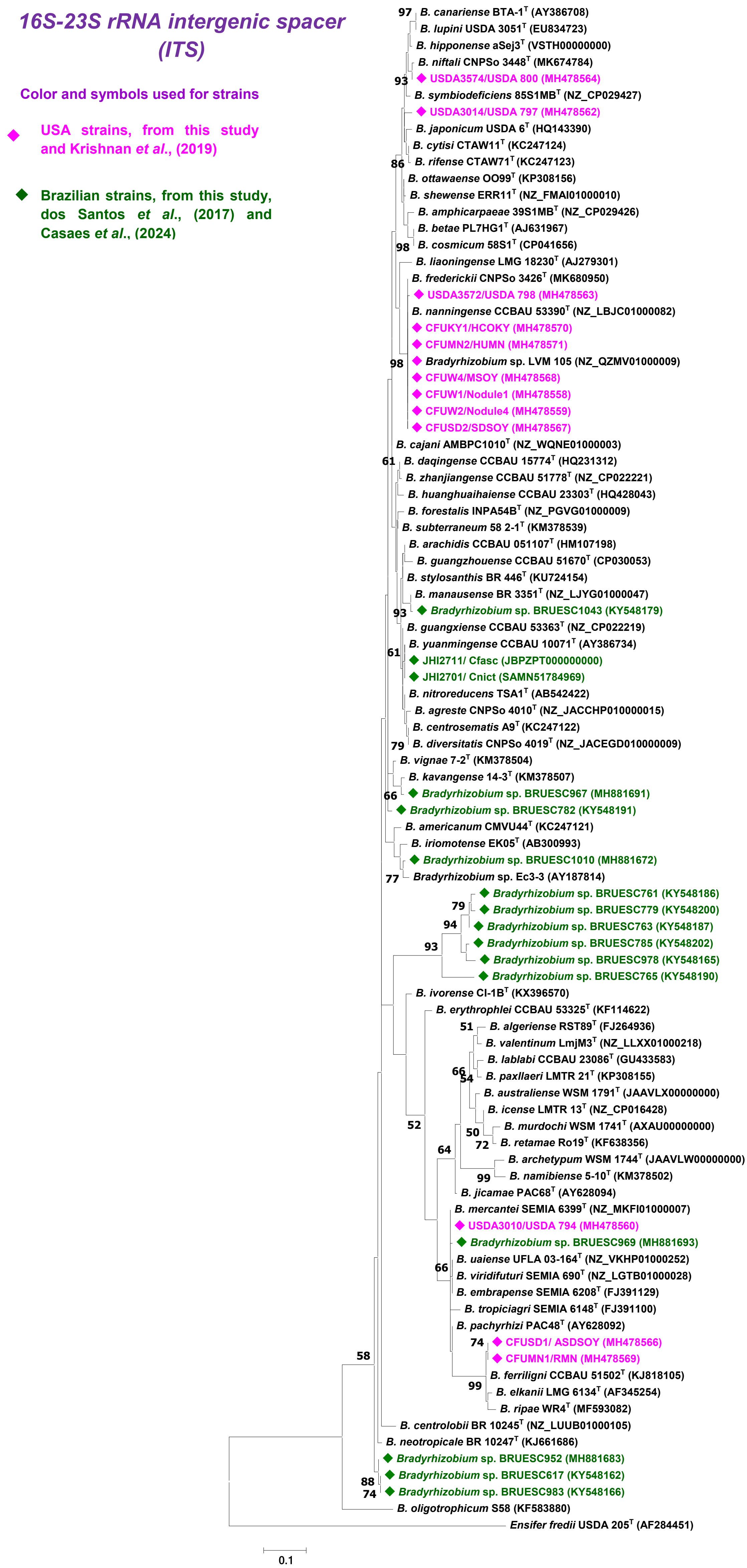

16S-23S rRNA intergenic spacer (ITS)
Color and symbols used for strains
USA strains, from this study and Krishnan et al., (2019)
Brazilian strains, from this study, dos Santos et al., (2017) and Casaes et al., (2024)
B. canariense BTA-1T (AY386708)
B. lupini USDA 3051T (EU834723)
B. hipponense aSej3T (VSTH00000000)
B. niftali CNPSo 3448T (MK674784)
USDA3574/USDA 800 (MH478564)
B. symbiodeficiens 85S1MBT (NZ_CP029427)
USDA3014/USDA 797 (MH478562)
B. japonicum USDA 6T (HQ143390)
B. cytisi CTAW11T (KC247124)
B. rifense CTAW71T (KC247123)
B. ottawaense OO99T (KP308156)
B. shewense ERR11T (NZ_FMAI01000010)
B. amphicarpaeae 39S1MBT (NZ_CP029426)
B. betae PL7HG1T (AJ631967)
B. cosmicum 58S1T (CP041656)
B. liaoningense LMG 18230T (AJ279301)
B. frederickii CNPSo 3426T (MK680950)
USDA3572/USDA 798 (MH478563)
B. nanningense CCBAU 53390T (NZ_LBJC01000082)
CFUKY1/HCOKY (MH478570)
CFUMN2/HUMN (MH478571)
Bradyrhizobium sp. LVM 105 (NZ_QZMV01000009)
CFUW4/MSOY (MH478568)
CFUW1/Nodule1 (MH478558)
CFUW2/Nodule4 (MH478559)
CFUSD2/SDSOY (MH478567)
B. cajani AMBPC1010T (NZ_WQNE01000003)
B. daqingense CCBAU 15774T (HQ231312)
B. zhanjiangense CCBAU 51778T (NZ_CP022221)
B. huanghuaihaiense CCBAU 23303T (HQ428043)
B. forestalis INPA54BT (NZ_PGVG01000009)
B. subterraneum 58 2-1T (KM378539)
B. arachidis CCBAU 051107T (HM107198)
B. guangzhouense CCBAU 51670T (CP030053)
B. stylosanthis BR 446T (KU724154)
B. manausense BR 3351T (NZ_LJYG01000047)
Bradyrhizobium sp. BRUESC1043 (KY548179)
B. guangxiense CCBAU 53363T (NZ_CP022219)
B. yuanmingense CCBAU 10071T (AY386734)
JHI2711/ Cfasc (JBPZPT000000000)
JHI2701/ Cnict (SAMN51784969)
B. nitroreducens TSA1T (AB542422)
B. agreste CNPSo 4010T (NZ_JACCHP010000015)
B. centrosematis A9T (KC247122)
B. diversitatis CNPSo 4019T (NZ_JACEGD010000009)
B. vignae 7-2T (KM378504)
B. kavangense 14-3T (KM378507)
Bradyrhizobium sp. BRUESC967 (MH881691)
Bradyrhizobium sp. BRUESC782 (KY548191)
B. americanum CMVU44T (KC247121)
B. iriomotense EK05T (AB300993)
Bradyrhizobium sp. BRUESC1010 (MH881672)
Bradyrhizobium sp. Ec3-3 (AY187814)
Bradyrhizobium sp. BRUESC761 (KY548186)
Bradyrhizobium sp. BRUESC779 (KY548200)
Bradyrhizobium sp. BRUESC763 (KY548187)
Bradyrhizobium sp. BRUESC785 (KY548202)
Bradyrhizobium sp. BRUESC978 (KY548165)
Bradyrhizobium sp. BRUESC765 (KY548190)
B. ivorense CI-1BT (KX396570)
B. erythrophlei CCBAU 53325T (KF114622)
B. algeriense RST89T (FJ264936)
B. valentinum LmjM3T (NZ_LLXX01000218)
B. lablabi CCBAU 23086T (GU433583)
B. paxllaeri LMTR 21T (KP308155)
B. australiense WSM 1791T (JAAVLX00000000)
B. icense LMTR 13T (NZ_CP016428)
B. murdochi WSM 1741T (AXAU00000000)
B. retamae Ro19T (KF638356)
B. archetypum WSM 1744T (JAAVLW00000000)
B. namibiense 5-10T (KM378502)
B. jicamae PAC68T (AY628094)
B. mercantei SEMIA 6399T (NZ_MKFI01000007)
USDA3010/USDA 794 (MH478560)
Bradyrhizobium sp. BRUESC969 (MH881693)
B. uaiense UFLA 03-164T (NZ_VKHP01000252)
B. viridifuturi SEMIA 690T (NZ_LGTB01000028)
B. embrapense SEMIA 6208T (FJ391129)
B. tropiciagri SEMIA 6148T (FJ391100)
B. pachyrhizi PAC48T (AY628092)
CFUSD1/ ASDSOY (MH478566)
CFUMN1/RMN (MH478569)
B. ferriligni CCBAU 51502T (KJ818105)
B. elkanii LMG 6134T (AF345254)
B. ripae WR4T (MF593082)
B. centrolobii BR 10245T (NZ_LUUB01000105)
B. neotropicale BR 10247T (KJ661686)
Bradyrhizobium sp. BRUESC952 (MH881683)
Bradyrhizobium sp. BRUESC617 (KY548162)
Bradyrhizobium sp. BRUESC983 (KY548166)
B. oligotrophicum S58 (KF583880)
Ensifer fredii USDA 205T (AF284451)
97
93
86
98
98
61
93
61
79
66
77
79
94
93
51
66
54
52
50
72
64
99
66
74
99
58
88
74
0.1

Fig. S1

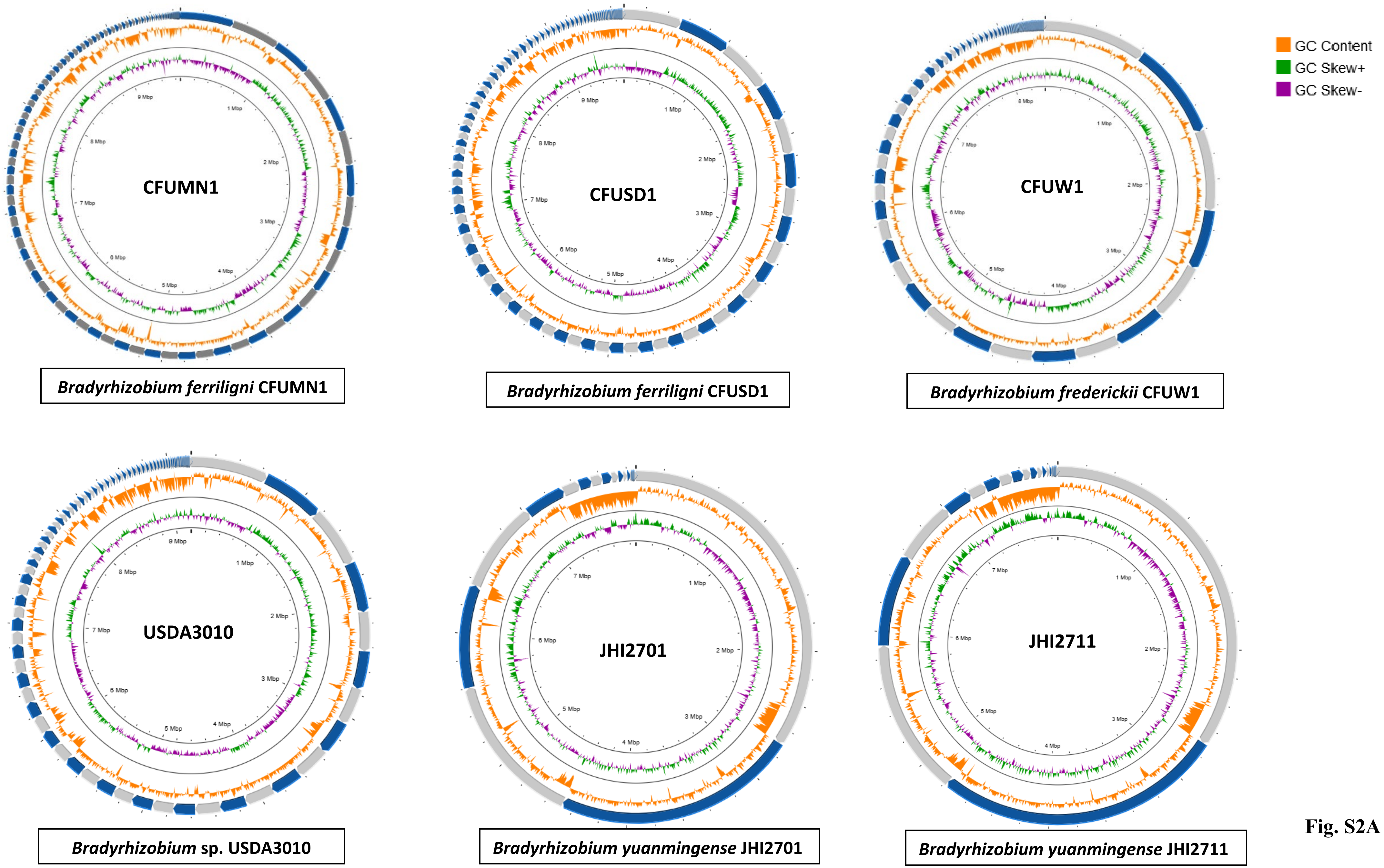
GC Content
GC Skew+
GC Skew-
CFUMN1
CFUSD1
CFUW1
USDA3010
JHI2701
JHI2711
Bradyrhizobium ferriligni CFUMN1
Bradyrhizobium ferriligni CFUSD1
Bradyrhizobium frederickii CFUW1
Bradyrhizobium sp. USDA3010
Bradyrhizobium yuanmingense JHI2701
Bradyrhizobium yuanmingense JHI2711

Fig. S2A

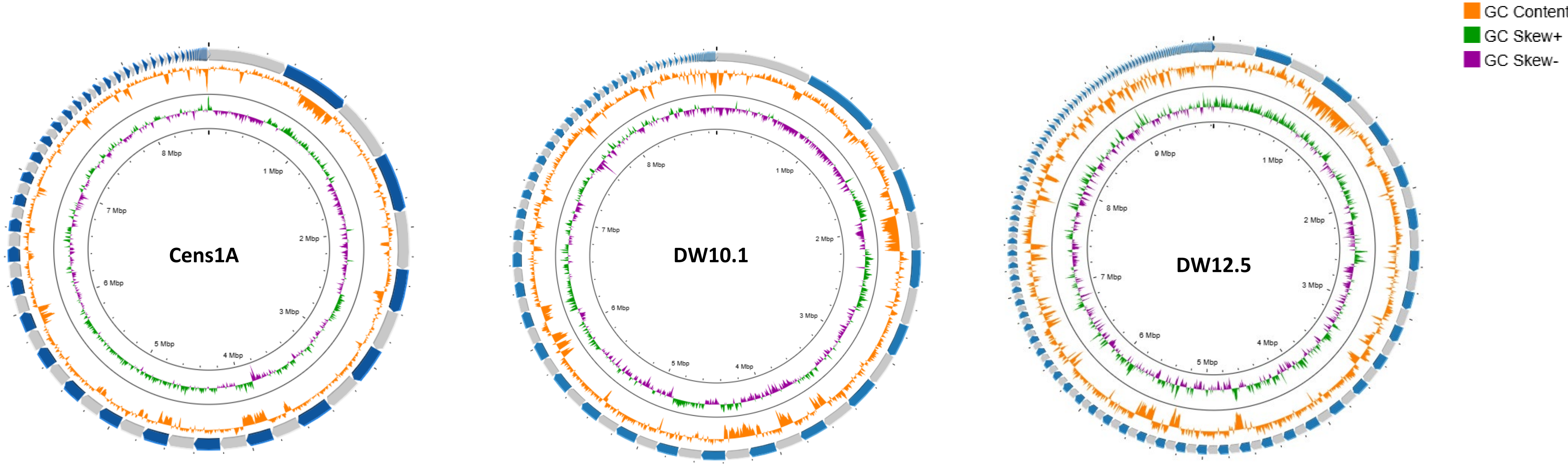

Bradyrhizobium sp. Cens1A

Bradyrhizobium sp. DW10.1

Bradyrhizobium sp. DW12.5

Fig. S2B

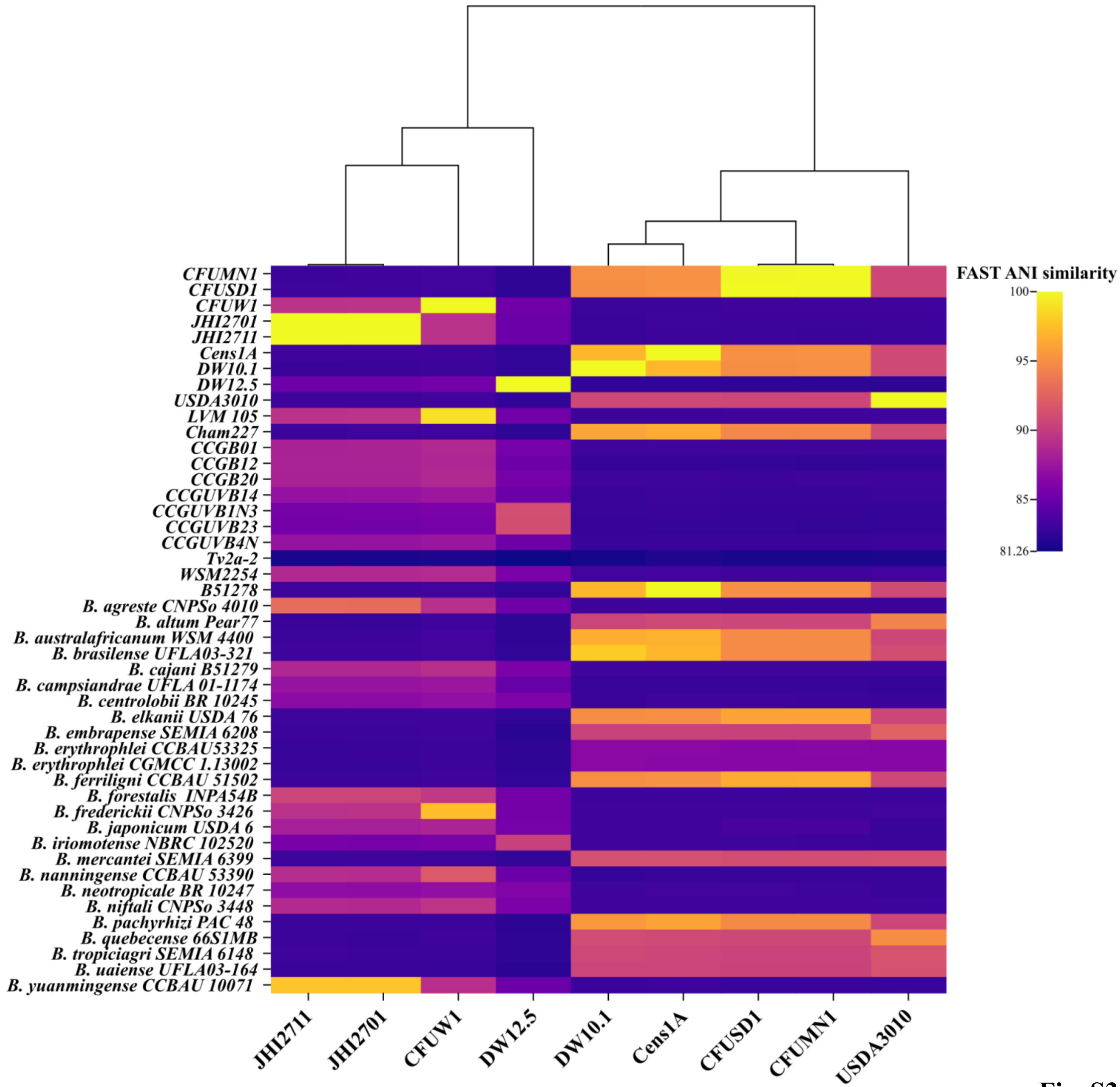
FAST ANI similarity
100
95
90
85
81.26
CFUMN1
CFUSD1
CFUW1
JHI2701
JHI2711
Cens1A
DW10.1
DW12.5
USDA3010
LVM 105
Cham227
CCGB01
CCGB12
CCGB20
CCGUVB14
CCGUVB1N3
CCGUVB23
CCGUVB4N
Tv2a-2
WSM2254
B51278
B. agreste CNPSo 4010
B. altum Pear77
B. australafricanum WSM 4400
B. brasilense UFLA03-321
B. cajani B51279
B. campsiandrae UFLA 01-1174
B. centrolobii BR 10245
B. elkanii USDA 76
B. embrapense SEMIA 6208
B. erythrophlei CCBAU53325
B. erythrophlei CGMCC 1.13002
B. ferriligni CCBAU 51502
B. forestalis INPA54B
B. frederickii CNPSo 3426
B. japonicum USDA 6
B. iriomotense NBRC 102520
B. mercantei SEMIA 6399
B. nanningense CCBAU 53390
B. neotropicale BR 10247
B. niftali CNPSo 3448
B. pachyrhizi PAC 48
B. quebecense 66S1MB
B. tropiciagri SEMIA 6148
B. uaiense UFLA03-164
B. yuanmingense CCBAU 10071
JHI2711
JHI2701
CFUW1
DW12.5
DW10.1
Cens1A
CFUSD1
CFUMN1
USDA3010

Fig. S3A

ANIb: Pair wise Average Nucleotide Identity (ANI) results based on BLAST+

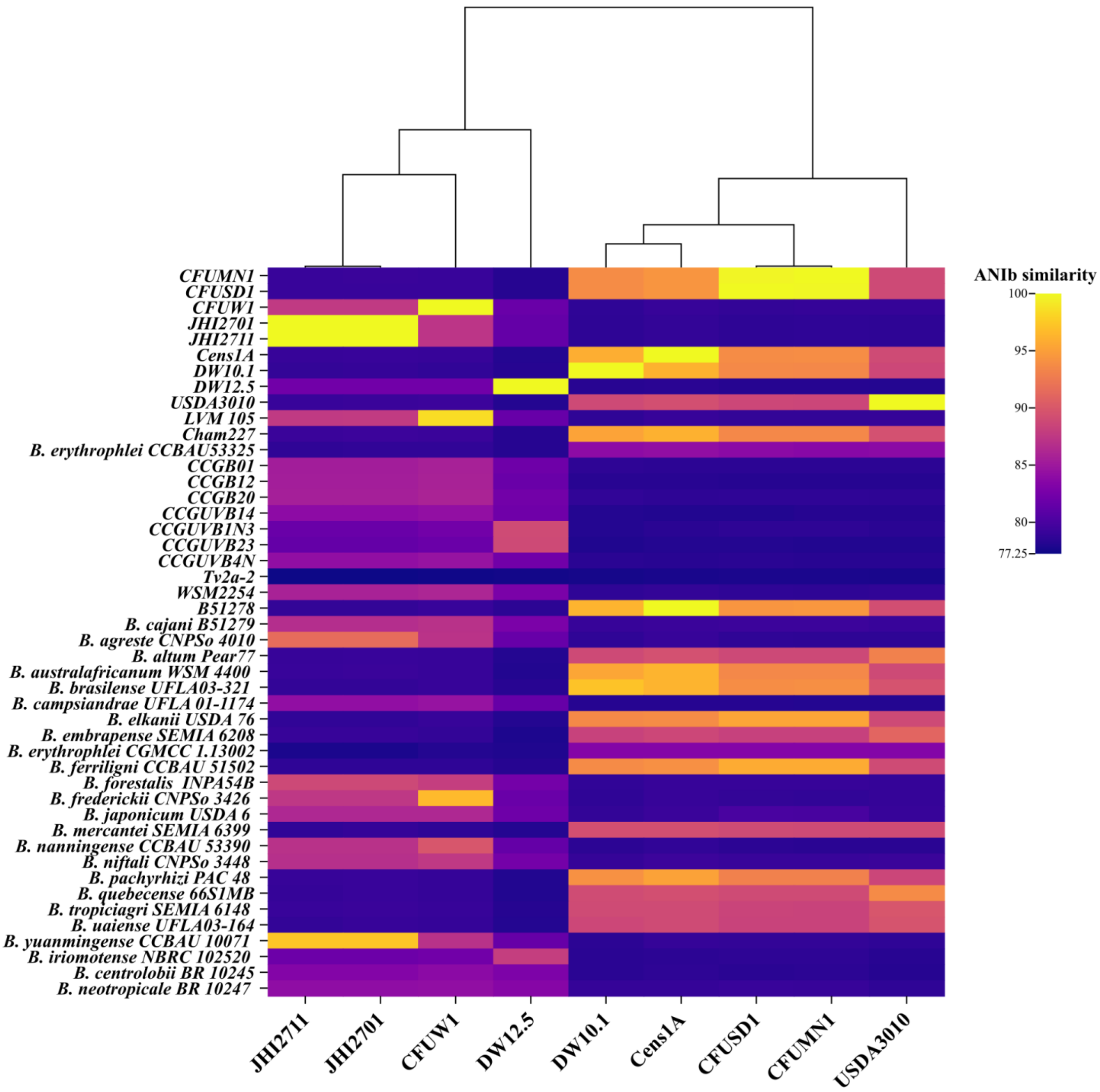

Fig. S3B

Genome BLAST Distance Phylogeny (GBDP) tree

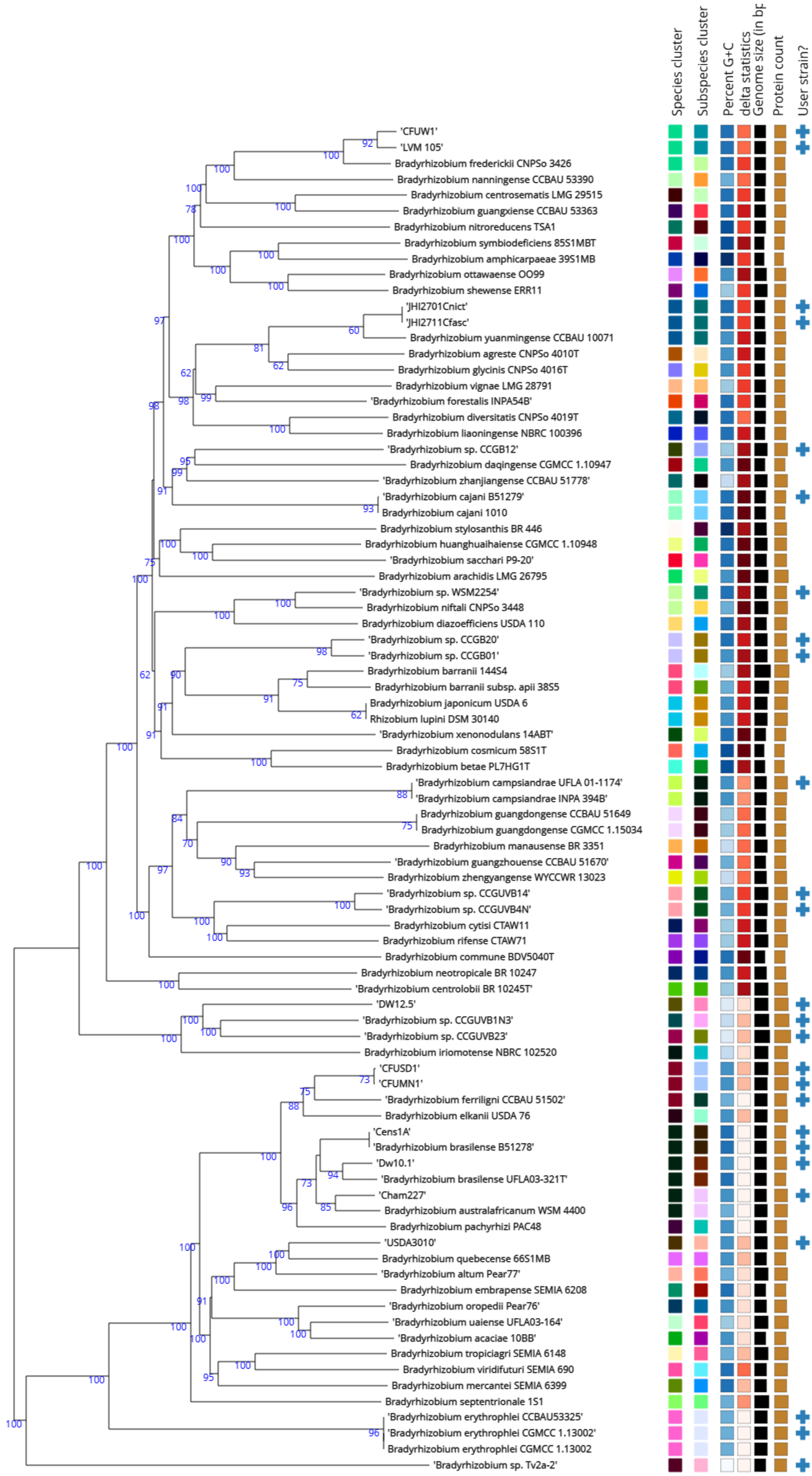

Fig. S4
