# Supplementary material for: Comparative genomic analysis of North American Bradyrhizobium symbionts of Chamaecrista fasciculata (Caesalpinioideae) with related bradyrhizobia
Source: Microbiol Spectr. 2026 Aug 19;14(10):e00798-26. doi: 10.1128/spectrum.00798-26 (PMC13689284; doi:10.1128/spectrum.00798-26)
Supplement: Supplementary file 2 [file spectrum.00798-26-s0002.pdf]

Table S1: List of primers and PCR cycling conditions used for molecular characterization of rhizobial strains

Primer	Oligonucleotide sequence* (5'→ 3')	Amplicon product name	Thermal cycling conditions	Reference
fD1<br>rD1	AGAGTTTGATCCTGGCTCAG<br>CTTAAGGAGGTGATCCAGCC	16S ribosomal RNA	5 min 95 °C, 30× (1 min 94 °C, 1 min 55 °C, 2 min 72 °C), 7 min 72 °C	Weisburg *et al.* (1991)
ITSF<br>ITSR	TCGGGCTGGATCACCTCCTT<br>CCGGGTTTCCCCATTCGG	16S-23S rRNA internal transcribed spacer	5 min 94 °C, 35× (1 min 94 °C, 1 min 55 °C, 1 min 72 °C), 7 min 72 °C	Dos Santos *et al*. (2017)
TSrecAF<br>TSrecAR	CAACTGCNYTGCGTATCGTCGAAGG<br>CGGATCTGGTTGATGAAGATCACCATG	Recombination protein	5 min 94 °C, 30× (45 s 94 °C, 1 min 57 °C, 1.5 min 72 °C), 7 min 72 °C	Stepkowski *et al.* (2005)
TSdnaK2<br>TSdnaK3	GTACATGGCCTCGCCGAGCTTCA<br>AAGGAGCAGCAGATCCGCATCCA	Molecular chaperone	5 min 95 °C, 35× (30 s 94 °C, 30 s 62 °C, 45 s 72 °C), 5 min 72 °C	Stepkowski *et al*. (2003)
nodAF<br>nodAR	TGCRGTGGAARNTRNNCTGGGAAA<br>GGNCCGTCRTCRAAWGTCARGTA	N-acyltransferase nodulation protein	2 min 95 °C, 30× (30s 95 °C, 30 s 60 °C, 1 min 72 °C), 5 min 72 °C	Haukka *et al.* (1998)
nifHF<br>nifHR	AARGGNGGNATYGGHAARTC<br>GCRTAVAKNGCCATCATYTC	Nitrogenase Fe protein	5 min 94 °C, 20× (30 s 94 °C, 30 s 60 °C, 1 min 72 °C), 25× (30 s 94 °C, 30 s 50 °C, 1 min 72 °C), 5 min 72 °C	Dos Santos *et al*. (2017)

* N= A, C, G or T; R= A or G; Y=C or T; K=G or T; V=A, C or G; W= A or T and S=C or G

Dos Santos JMF, Alves PAC, Silva VC, Rhem MFK, James EK, Gross E. 2017. Diverse genotypes of *Bradyrhizobium* nodulate herbaceous *Chamaecrista* (Moench) (Fabaceae, Caesalpinioideae) species in Brazil. Syst Appl Microbiol 40:69–79.

Haukka K, Lindström K, Young JPW. 1998. Three phylogenetic groups of *nodA* and *nifH* genes in *Sinorhizobium* and *Mesorhizobium* isolates from leguminous trees growing in Africa and Latin America. Appl Environ Microbiol 64:419–426.

Stępkowski T, Czaplińska M, Miedzinska K, Moulin L. 2003. The variable part of the *dnaK* gene as an alternative marker for phylogenetic studies of rhizobia and related alpha Proteobacteria. Syst Appl Microbiol 26:483–494.

Stępkowski T, Moulin L, Krzyżańska A, McInnes A, Law IJ, Howieson J. 2005. European origin of *Bradyrhizobium* populations infecting lupins and serradella in soils of Western Australia and South Africa. Appl Environ Microbiol 71:7041–7052.

Weisburg WG, Barns SM, Pelletier DA, Lane DJ. 1991. 16S ribosomal DNA amplification for phylogenetic study. J Bacteriol 173:697–703.

Table S2: GenBank accesion numbers for intergenic spacer, housekeeping gene, symbiotic gene sequences and genome assemblies of *Bradyrhizobium* strains

Strain name	Earlier/Other name	Biosample accession	NCBI accession numbers	16S rRNA (*rrs*)	16S-23S rRNA intergenic spacer (ITS)	*recA*	*dnaK*	*nodA*	*nifH*
**CFUW1**	Nodule 1	SAMN51784967	JBWJZE000000000	MH094863	MH478558	MH760506	MK310155	MH988702	MK314836
CFUW2	Nodule 4	-		MH470238	MH478559	MH760507	MK310156	MH988703	MK314837
CFUW3	Nodule ZZ1	-		MH102347	MH478565	MH760512	MK310161	MH988707	MK314842
CFUW4	MSOY	-		MH102352	MH478568	MH760515	MK310164	MH988710	MK314845
**CFUSD1**	ASDSOY	SAMN51784965	JBWJZG000000000	MH102351	MH478566	MH760513	MK310162	MH988708	MK314843
CFUSD2	SDSOY	-		MH102350	MH478567	MH760514	MK310163	MH988709	MK314844
**CFUMN1**	RMN	SAMN51784966	JBWJZF000000000	MH102353	MH478569	MH760516	MK310165	MH988711	MK314846
CFUMN2	HUMN	-		MH102348	MH478571	MH760517	MK310166	MH988712	MK314847
CFUKY1	HCOKY	-		MH102349	MH478570	MH760518	MK310167	MH988713	MK314848
**USDA3010**	USDA794	SAMN51784968	JBWJZD000000000	MH102344	MH478560	MH760508	MK310157	MK514142	MK314838
USDA3014	USDA797	-		MK118922	MH478562	MH760509	MK310158	MK514143	MK314839
USDA3572	USDA798	-		MH102345	MH478563	MH760510	MK310159	MH988705/ MT814322	MK314840
USDA3574	USDA800	-		MH102346	MH478564	MH760511	MK310160	MH988706	MK314841
**JHI2701**	Cnict	SAMN51784969	JBWJZC000000000						
**JHI2711**	Cfasc	SAMN50045062	JBPZPT000000000						
**Cens1A**	BR3804A	SAMN50045061	JBPZPU000000000						
**DW10.1**	-	SAMN50045063	JBPZPS000000000						
**DW12.5**	-	SAMN50045064	JBPZPR000000000						
**CCBAU 53325^T**	JHI1531	SAMN50045065	JBPZPQ000000000						
**CCBAU 51502^T**	JHI1527	SAMN50045066							

Table S3: Summary of Illumina next-generation sequencing (NGS) data. The reads were trimmed using Trimmomatic and the quality was assessed using in-house scripts of MicobesNG combined with the following softwares: SAM (Sequence Alignment/Map) Samtools, (Browser Extensible Data) BedTools and bwa-mem

Strain name	Barcode/sample ID	Coverage	Median insert size	Mean coverage	Mean coverage excluding 0s	Number of reads	Number of reads w/ insert size > 300	Genome coverage
CFUW1	14503_BradyrhizobiumCFUW1	30x	-	119.133	119.133	2329706	-	30
CFUSD1	31305_JHI2704ASDSOY	30x	711	43.2529	43.2647	885737	729639	43.2529
CFUMN1	31306_JHI2709RMN	30x	712	53.5368	53.5476	1097091	885355	53.5368
USDA3010	31304_JHI2702USDA3010	30x	736	58.1105	58.1277	1133140	893748	58.1105
JHI2701	31303_JHI2701	30x	744	62.7611	62.7694	1049977	805124	62.7611
JHI2711	31307_JHI2711Cfasc	30x	778	79.2021	79.2105	1325668	1040350	79.2021
Cens1A	231701_Cens1A	30x	677	112.936	112.959	2025297	1771482	112.936
DW10.1	Microbes NG: 10704	30x	463	77.1038	77.1047	1714395	1138387	47.1653
DW12.5	Microbes NG: 10705	30x	-	-	-	-	-	38.721
CCBAU53325	231675_Bradyrhizobium erythrophlei CCBAU53325T	30x	650	56.3436	56.3749	1169199	1043905	56.3436
CCBAU 51502	11387_Bradyrhizobium ferriligni	30x	94	57.5604	57.5688	2543329	19068	57.5604

Table S4: The genome assembly metrics calculated using QUAST a Quality Assessment Tool for Genome Assemblies by CAB (Center for Algorithmic Biotechnology)

Assembly statistics	**CFUW1**	**CFUSD1**	**CFUMN1**	**USDA3010**	**JHI2701**	**JHI2711**	**Cens1A**	**DW10.1**	**DW12.5**	**CCBAU53325**	**CCBAU 51502**
# contigs	152	169	132	138	26	27	103	125	286	223	230
# contigs (>= 0 bp)	365	245	205	213	55	69	175	237	328	414	629
# contigs (>= 1000 bp)	112	152	120	128	21	25	98	111	252	193	186
# contigs (>= 5000 bp)	67	115	101	98	16	18	77	91	190	150	146
# contigs (>= 10000 bp)	55	100	94	89	15	17	72	82	159	132	132
# contigs (>= 25000 bp)	41	77	80	62	14	15	66	67	106	99	105
# contigs (>= 50000 bp)	28	57	56	42	10	11	42	48	64	66	73
Largest contig	825427	536494	484671	655189	2708515	2708515	551758	685420	331763	498255	224858
Total length	8286627	9699723	9649768	9181251	7861681	7860189	8575174	8844051	9820018	9505889	9144460
Total length (>= 0 bp)	8373749	9718390	9667046	9200487	7869077	7869294	8599188	8889837	9832845	9555590	9268296
Total length (>= 1000 bp)	8260262	9687871	9641723	9173647	7858022	7858759	8571597	8834215	9794403	9485568	9114570
Total length (>= 5000 bp)	8163237	9610062	9607845	9102744	7848749	7846427	8523111	8792700	9655002	9399622	9014593
Total length (>= 10000 bp)	8076284	9498295	9558184	9040648	7842564	7840242	8485786	8732247	9432347	9270147	8905560
Total length (>= 25000 bp)	7836592	9085052	9311499	8588873	7832440	7815364	8387527	8459066	8566624	8670266	8439457
Total length (>= 50000 bp)	7392362	8326886	8390265	7889539	7692142	7674696	7527199	7747197	6980087	7497560	7244533
N50	350136	134722	160979	183963	1775508	2075325	184416	170399	84328	100509	92331
N75	131610	77954	91881	113530	758045	1118741	108843	105348	47434	59486	54954
L50	8	19	19	14	2	2	14	16	33	28	35
L75	17	41	39	29	4	3	28	32	72	59	66
GC (%)	63.92	63.67	63.71	63.66	63.92	63.92	63.97	63.83	62.49	63.43	63.68

All statistics are based on contigs of size >= 500 bp, unless otherwise noted (e.g., "# contigs (>= 0 bp)" and "Total length (>= 0 bp)" include all contigs).

Table S5: Rapid assessment of genome bin quality using machine learning through checkm2 (Galaxy Version 1.1.0+galaxy0)

Name	Completeness	Contamination	Completeness Model Used	Translation Table Used	Coding Density	Contig N50	Average Gene Length	Genome Size	GC Content	Total Coding Sequences	Total Contigs	Max Contig Length
CFUW1	100	0.93	Neural Network (Specific Model)	11	0.842	350136	294.7793	8373749	0.64	7988	365	825427
CFUSD1	100	3.65	Neural Network (Specific Model)	11	0.833	134722	293.3365	9718390	0.64	9215	245	536494
CFUMN1	100	4.2	Neural Network (Specific Model)	11	0.836	160979	294.2348	9667046	0.64	9172	205	484671
USDA3010	100	0.44	Neural Network (Specific Model)	11	0.842	183963	297.2758	9200487	0.64	8694	213	655189
JHI2701	100	0.83	Neural Network (Specific Model)	11	0.853	1775508	306.6161	7869077	0.64	7309	55	2708515
JHI2711	100	0.83	Neural Network (Specific Model)	11	0.853	2075325	306.1933	7869294	0.64	7320	69	2708515
Cens1A	100	0.68	Neural Network (Specific Model)	11	0.846	184416	308.4896	8599188	0.64	7868	175	551758
DW10.1	100	2.81	Neural Network (Specific Model)	11	0.843	170399	302.4819	8889837	0.64	8271	237	685420
DW12.5	100	5.01	Neural Network (Specific Model)	11	0.831	84328	285.8613	9832845	0.62	9550	328	331763
CCBAU53325	100	2.68	Neural Network (Specific Model)	11	0.838	94415	292.2166	9555590	0.63	9151	414	498255
CCBAU 51502	100	1.37	Neural Network (Specific Model)	11	0.831	92013	299.7111	9268296	0.63	8573	629	224858

Table S6: Details about the genome sequences used in present study for whole genome based phylogenetic tree

TYGS ID	Kind	Preferred name	Deposit	Authority	Other deposits	Base pairs	Percent G+C	No. proteins	Bioproject accession	Biosample accession	Assembly accession
U989441	user strain	CFUMN1	NA	This study		9,667,046	63.71	9172	PRJNA1293807	SAMN51784966	JBWJZF000000000
U989443	user strain	CFUSD1	NA	This study		9,718,390	63.67	9215	PRJNA1293807	SAMN51784965	JBWJZG000000000
U989444	user strain	CFUW1	NA	This study		8,373,749	63.92	7988	PRJNA1293807	SAMN51784967	JBWJZE000000000
U989442	user strain	JHI2701	NA	This study		7,869,077	63.92	7309	PRJNA1293807	SAMN51784969	JBWJZC000000000
U989439	user strain	JHI2711	NA	This study		7,869,294	63.92	7320	PRJNA1293807	SAMN50045062	JBPZPT000000000
U989435	user strain	Cens1A	NA	This study		8,599,188	63.95	7868	PRJNA1293807	SAMN50045061	JBPZPU000000000
U989419	user strain	DW10.1	NA	This study		8,889,837	63.8	8271	PRJNA1293807	SAMN50045063	JBPZPS000000000
U989429	user strain	DW12.5	NA	This study		9,832,845	62.48	9550	PRJNA1293807	SAMN50045064	JBPZPR000000000
U989440	user strain	USDA3010	NA	This study		9,200,487	63.65	8694	PRJNA1293807	SAMN51784968	JBWJZD000000000
U989436	user/type strain	*Bradyrhizobium erythrophlei* CCBAU53325	CCBAU 53325T	Yao et al. 2015		9,555,590	63.42	9151	PRJNA1293807	SAMN50045065	JBPZPQ000000000
U989437	user/type strain	*Bradyrhizobium ferriligni* CCBAU 51502	CCBAU 51502T	Yao et al. 2015		9,268,296	63.37	8573	PRJNA1293807	SAMN50045066	
U989438	user strain	LVM 105	NA	University of Missouri		8,326,213	63.88	7789	PRJNA492229	SAMN10095282	GCF_003957705
U989434	user strain	Cham227		University of Pretoria		9,072,283	63.71	8997	PRJNA657394	SAMN15826093	GCF_020889715
U989420	user strain	*Bradyrhizobium sp.* CCGB01		Universidad Nacional Autonoma de Mexico		9,317,328	63.79	8730	PRJNA842432	SAMN28667590	GCF_024199795
U989424	user strain	*Bradyrhizobium sp.* CCGB12		Universidad Nacional Autonoma de Mexico		9,426,670	63.11	8690	PRJNA842432	SAMN28667586	GCF_024199845
U989423	user strain	*Bradyrhizobium sp.* CCGB20		Universidad Nacional Autonoma de Mexico		9,227,337	63.84	8622	PRJNA842432	SAMN28667587	GCF_024199855
U989428	user strain	*Bradyrhizobium sp.* CCGUVB14		Universidad Nacional Autonoma de Mexico		9,769,664	63.3	9123	PRJNA842432	SAMN28667582	GCF_024199935
U989427	user strain	*Bradyrhizobium sp.* CCGUVB1N3		Universidad Nacional Autonoma de Mexico		10,444,098	62.88	9915	PRJNA842432	SAMN28667583	GCF_024199925
U989426	user strain	*Bradyrhizobium sp.* CCGUVB23		Universidad Nacional Autonoma de Mexico		11,273,073	62.54	10778	PRJNA842432	SAMN28667584	GCF_024199885
U989425	user strain	*Bradyrhizobium sp.* CCGUVB4N		Universidad Nacional Autonoma de Mexico		9,212,720	63.56	8526	PRJNA842432	SAMN28667585	GCF_024199945
U989422	user strain	*Bradyrhizobium brasilense* B51278		Universidad Nacional Autonoma de Mexico		8,566,773	63.98	7799	PRJNA842432	SAMN28667588	GCF_024199825
U989421	user strain	*Bradyrhizobium cajani* B51279		Universidad Nacional Autonoma de Mexico		8,361,402	63.97	7696	PRJNA842432	SAMN28667589	GCF_024199785
U989432	user strain	*Bradyrhizobium sp.* Tv2a-2		DOE Joint Genome Institute		8,496,279	62.2	8098	PRJNA165315	SAMN02440785	GCF_000472425
U989431	user strain	*Bradyrhizobium sp.* WSM2254		DOE Joint Genome Institute		9,086,516	63.88	8517	PRJNA165519	SAMN02440783	GCF_000472645
U989433	user strain	*Bradyrhizobium campsiandrae'* UFLA 01-1174	INPA 394B	Cabral Michel et al. 2021	LMG 10099; INPA 394B	9,084,813	63.63	8761	PRJNA224116	SAMN14291063	GCF_014529705
	user strain	*Bradyrhizobium erythrophlei* CGMCC 1.13002	CGMCC 1.13002	Yao et al. 2015	CCBAU 53325; HAMBI 3614;	9,470,608	63.44	8939	PRJNA595761	SAMN43281877	GCF_042430365
122078	type strain	*'Bradyrhizobium acaciae'*	10BB	Avontuur et al. 2022	LMG 31409; SARCC 730	8,467,363	63.69	7728	PRJNA657394	SAMN15826096	GCA_020889785
92652	type strain	*Bradyrhizobium agreste*	CNPSo 4010T	Klepa et al. 2021	LMG 31645; WSM 4802	7,871,244	63.81	7273	PRJNA224116	SAMN15495217	GCF_016031625
93235	type strain	*'Bradyrhizobium altum'*	Pear77	Avontuur et al. 2022	LMG 31407; SARCC 754	9,749,879	63.32	8843	PRJNA657394	SAMN15826095	GCA_020889705
13557	type strain	*Bradyrhizobium amphicarpaeae*	39S1MB	Bromfield et al. 2019	HAMBI 3680; LMG 29934	7,044,517	64.66	6441	PRJNA397110	SAMN07448754	GCA_002266435
13825	type strain	*Bradyrhizobium arachidis*	LMG 26795	Wang et al. 2015 emend. Hördt et al. 2020	CGMCC 1.12100; CCBAU 51107; HAMBI 3281	9,792,874	63.63	9312	PRJEB17582	SAMN05192541	GCA_900116675
70981	type strain	*Bradyrhizobium australafricanum*	WSM 4400	Klepa et al. 2022	CNPSo 4015; LMG 31648	9,681,271	63.52	8860	PRJNA717299	SAMN18497705	GCA_020329495
71334	type strain	*Bradyrhizobium barranii*	144S4	Bromfield et al. 2022	HAMBI 3722; LMG 31552	11,399,526	63.13	10154	PRJNA714589	SAMN18312838	GCA_017565645
120074	type strain	*Bradyrhizobium barranii subsp. apii*	38S5	Bromfield et al. 2022	HAMBI 3721; LMG 31556	10,530,141	63.34	9234	PRJNA714594	SAMN18312895	GCA_017565685
15607	type strain	*Bradyrhizobium betae*	PL7HG1T	Rivas et al. 2004	CECT 5829; DSM 17288; NBRC 103048; LMG 21987	7,419,402	64.36	6780	PRJNA575551	SAMN12900461	GCA_008932115
9523	type strain	*'Bradyrhizobium brasilense'*	UFLA03-321T	Martins da Costa et al. 2017	CBAS 645; LMG 29353	8,595,048	63.93	7632	PRJNA354699	SAMN06050723	GCA_001969825
**TYGS ID**	**Kind**	**Preferred name**	**Deposit**	**Authority**	**Other deposits**	**Base pairs**	**Percent G+C**	**No. proteins**	**Bioproject accession**	**Biosample accession**	**Assembly accession**
17297	type strain	*Bradyrhizobium cajani*	1010	Araújo et al. 2017	AMBPC1010; CECT 9227; LMG 29967	8,372,827	63.96	7348	PRJNA593773	SAMN13489115	GCA_009759665
131991	type strain	*'Bradyrhizobium campsiandrae'*	INPA 394B	Cabral Michel et al. 2021	LMG 10099; UFLA 01-1174T	9,051,172	63.65	8467	PRJNA610245	SAMN14291063	GCA_014529705
10827	type strain	*'Bradyrhizobium centrolobii'*	BR 10245T	Michel et al. 2017	HAMBI 3597	10,106,545	63.22	8821	PRJNA301972	SAMN04448272	GCA_001641635

6579	type strain	*Bradyrhizobium centrosematis*	LMG 29515	Ramírez-Bahena et al. 2017	A9; CECT 9095	7,598,687	64.05	7095	PRJNA944954	SAMN33769197	GCA_045571495
115578	type strain	*Bradyrhizobium commune*	BDV5040T	Lafay et al. 2023	CFBP 9110; LMG 32898	7,622,528	63.92	7107	PRJNA224116	SAMN16089659	GCF_015624505
41138	type strain	*Bradyrhizobium cosmicum*	58S1T	Wasai-Hara et al. 2020	HAMBI 3725; LMG 31545	7,304,136	64.33	6924	PRJNA224116	SAMN12262001	GCF_007290395
18513	type strain	*Bradyrhizobium cytisi*	CTAW11	Chahboune et al. 2011	CECT 7749; DSM 23720; LMG 25866	8,849,532	63.17	7900	PRJNA560796	SAMN12600325	GCA_008123515
16415	type strain	*Bradyrhizobium daqingense*	CGMCC 1.10947	Wang et al. 2013	CCBAU 15774; HAMBI 3184; LMG 26137	7,885,317	63.74	7442	PRJNA255603	SAMN02927912	GCA_007830205
2697	type strain	*Bradyrhizobium diazoefficiens*	USDA 110	Delamuta et al. 2013	3I1B110; ACCC 15034; CNPSo 46; BCRC 13528; CCRC 13528; NRRL B-4361; NRRL B-4450; JCM 10833; IFO 14792; NBRC 14792; CCT 4249; IAM 13628; R-12974; SEMIA 5032; TAL 102; TISTR 339	9,105,828	64.06	8317	PRJNA17	SAMD00061083	GCA_000011365
92658	type strain	*Bradyrhizobium diversitatis*	CNPSo 4019T	Klepa et al. 2021	LMG 31650; WSM 4799	8,437,014	63.93	7915	PRJNA224116	SAMN15581659	GCF_016031635
5147	type strain	*Bradyrhizobium elkanii*	USDA 76	Kuykendall et al. 1993 emend. Hördt et al. 2020	ATCC 49852; DSM 11554; IFO 14791; NBRC 14791; LMG 6134	9,482,180	63.72	8962	PRJNA162247	SAMN02261261	GCA_000379145
4358	type strain	*Bradyrhizobium embrapense*	SEMIA 6208	Delamuta et al. 2015 emend. Hördt et al. 2020	CNPSo 2833; BR 2212; CIAT 2372; LMG 29087; U674	8,267,800	63.98	7721	PRJNA287423	SAMN03782074	GCA_001189235
2081	type strain	*Bradyrhizobium erythrophlei*	CGMCC 1.13002	Yao et al. 2015	CCBAU 53325; HAMBI 3614; LMG 28425	9,457,018	63.44	8668	PRJNA595761	SAMN43281877	GCA_042430365
15620	type strain	*'Bradyrhizobium forestalis'*	INPA54B	Martins da Costa et al. 2018	LMG 10044	8,235,644	63.87	7360	PRJNA418781	SAMN08035463	GCA_002795245
14515	type strain	*Bradyrhizobium frederickii*	CNPSo 3426	de Oliveira Urquiaga et al. 2019	CL 20; U686; USDA 10052	8,291,487	63.85	7506	PRJNA529073	SAMN11254746	GCA_004570865
126706	type strain	*Bradyrhizobium glycinis*	CNPSo 4016T	Klepa et al. 2021	LMG 31649; WSM 4801	7,790,296	63.73	7322	PRJNA224116	SAMN15496426	GCF_016031655
48416	type strain	*Bradyrhizobium guangdongense*	CGMCC 1.15034	Li et al. 2015	CGMCC 1.15034; CCBAU 51649; LMG 28620	8,345,782	63.26	8032	PRJDB10509	SAMD00245008	GCA_014640515
6945	type strain	*Bradyrhizobium guangdongense*	CCBAU 51649	Li et al. 2015	CGMCC 1.15034; CCBAU 51649; LMG 28620	8,437,991	63.26	7648	PRJNA229440	SAMN02415993	GCA_004114975
17916	type strain	*Bradyrhizobium guangxiense*	CCBAU 53363	Li et al. 2015	CGMCC 1.15035; LMG 28261; LMG 28621	8,200,121	63.91	7796	PRJNA229447	SAMN02418808	GCA_004114915
13980	type strain	*'Bradyrhizobium guangzhouense'*	CCBAU 51670	Li et al. 2019	HAMBI 3650; LMG 29280	8,138,177	63.39	7653	PRJNA224116	SAMN03459144	GCF_004114955
18506	type strain	*Bradyrhizobium huanghuaihaiense*	CGMCC 1.10948	Zhang et al. 2012	CCBAU 23303; HAMBI 3180; LMG 26136	9,223,029	63.94	8622	PRJNA255602	SAMN02927902	GCA_007830635
94558	type strain	*Bradyrhizobium iriomotense*	NBRC 102520	Islam et al. 2010	EK05; LMG 24129	9,736,893	62.9	9092	PRJDB15044	SAMD00576799	GCA_030160715
339	type strain	*Bradyrhizobium japonicum*	USDA 6	(Kirchner 1896) Jordan 1982 emend. Hördt et al. 2020	CIP 106093; NRRL B-4507; NRRL L-241; ATCC 10324; CCUG 27876; DSM 30131; JCM 20679; IFO 14783; NBRC 14783; VKM B-1967; HAMBI 2314; LMG 6138	9,207,384	63.67	8829	PRJDA67463	SAMD00060992	GCA_000284375
**TYGS ID**	**Kind**	**Preferred name**	**Deposit**	**Authority**	**Other deposits**	**Base pairs**	**Percent G+C**	**No. proteins**	**Bioproject accession**	**Biosample accession**	**Assembly accession**
115474	type strain	*Bradyrhizobium liaoningense*	NBRC 100396	Xu et al. 1995	2281; CIP 104858; ATCC 700350; DSM 24092; LMG 18230	7,830,169	63.95	7615	PRJDB15044	SAMD00576800	GCA_030160735
15667	type strain	*Rhizobium lupini*	DSM 30140	(Schroeter 1886) Eckhardt et al. 1931	3C231; BCRC 17460; CCRC 17460; ATCC 10319; JCM 20681; NBRC 100381; VKM B-1965; IAM 12610	9,125,394	63.7	8822			
3843	type strain	*Bradyrhizobium manausense*	BR 3351	Silva et al. 2014	HAMBI 3596	9,133,688	62.87	7794	PRJNA295814	SAMN04088902	GCA_001440035
23387	type strain	*Bradyrhizobium mercantei*	SEMIA 6399	Helene et al. 2017	CNPSo 1165; BR 6010; LMG 30031; U675	8,842,806	63.99	8096	PRJNA344479	SAMN05826124	GCA_001982635
19743	type strain	*Bradyrhizobium nanningense*	CCBAU 53390	Li et al. 2020	HAMBI 3651; LMG 29278	8,284,532	63.44	7716	PRJNA224116	SAMN03459137	GCF_004114535
3745	type strain	*Bradyrhizobium neotropicale*	BR 10247	Zilli et al. 2014	HAMBI 3599	8,676,530	63.6	7682	PRJNA311013	SAMN04457964	GCA_001641695
18779	type strain	*Bradyrhizobium niftali*	CNPSo 3448	Klepa et al. 2019	CL 40; U687; USDA 10051	9,786,055	63.53	8664	PRJNA529074	SAMN11254745	GCA_004571025

8305	type strain	*Bradyrhizobium nitroreducens*	TSA1	Jang et al. 2020	KCTC 62391; ATCC TSD-124; JCM 18858	8,197,857	64.31	7268	PRJNA286791	SAMN03770688	GCA_002776695
93234	type strain	*'Bradyrhizobium oropedii'*	Pear76	Avontuur et al. 2022	LMG 31408; SARCC 731	9,026,748	63.57	8155	PRJNA657394	SAMN15826094	GCA_020889685
23426	type strain	*Bradyrhizobium ottawaense*	OO99	Yu et al. 2014 emend. Hördt et al. 2020	HAMBI 3284; LMG 26739	8,677,089	63.82	7774	PRJNA397101	SAMN07448707	GCA_002278135
4490	type strain	*Bradyrhizobium pachyrhizi*	PAC48	Ramírez-Bahena et al. 2009	CECT 7396; DSM 19631; LMG 24246	8,706,184	63.76	8213	PRJNA287433	SAMN03782120	GCA_001189245
118248	type strain	*Bradyrhizobium quebecense*	66S1MB	Bromfield and Cloutier 2021	HAMBI 3720; LMG 31547	9,032,145	63.82	7999	PRJNA640100	SAMN15299083	GCA_013373795
10736	type strain	*Bradyrhizobium rifense*	CTAW71	Chahboune et al. 2014	CECT 8066; LMG 26781	9,952,701	63.02	8975	PRJNA560799	SAMN12600330	GCA_008123425
14517	type strain	*'Bradyrhizobium sacchari'*	P9-20	de Matos et al. 2017	BR 10280; HAMBI 3667	8,685,818	63.84	8163	PRJNA224116	SAMN04849207	GCF_002068095
118247	type strain	*Bradyrhizobium septentrionale*	1S1	Bromfield and Cloutier 2021	HAMBI 3676; LMG 29930	10,598,256	63.49	9227	PRJNA612041	SAMN14360499	GCA_011516645
18997	type strain	*Bradyrhizobium shewense*	ERR11	Aserse et al. 2018	HAMBI 3532; LMG 30162	9,162,066	63.23	8018	PRJNA224116	SAMN04487809	GCF_900094605
6257	type strain	*Bradyrhizobium stylosanthis*	BR 446	Delamuta et al. 2016	CNPSo 2823; H-8; HAMBI 3668	8,801,645	64.56	8141	PRJNA315153	SAMN04549886	GCA_001641335
8818	type strain	*Bradyrhizobium symbiodeficiens*	85S1MBT	Bromfield et al. 2020	HAMBI 3684; LMG 29937	7,039,503	64.27	6585	PRJNA224116	SAMN07448748	GCF_002266465
5113	type strain	*Bradyrhizobium tropiciagri*	SEMIA 6148	Delamuta et al. 2015	CNPSo 1112; BR 1009; LMG 28867; SMS 303	9,767,314	63.49	9348	PRJNA287624	SAMN03784761	GCA_001189845
44556	type strain	*'Bradyrhizobium uaiense'*	UFLA03-164	Cabral Michel et al. 2020	LMG 31509	9,868,029	63.27	9162	PRJNA418781	SAMN12237784	GCA_010811875
9288	type strain	*Bradyrhizobium vignae*	LMG 28791	Grönemeyer et al. 2016	7-2; DSM 100297; NTCCM0018(Windhoek); NTCCM 18	8,169,430	63.22	7297	PRJNA497496	SAMN10259397	GCA_004114425
4363	type strain	*Bradyrhizobium viridifuturi*	SEMIA 690	Helene et al. 2015	CNPSo 991; BR 1804; C 100a; LMG 28866	8,811,922	64.03	8790	PRJNA290320	SAMN03890369	GCA_001238275
104516	type strain	*'Bradyrhizobium xenonodulans'*	14ABT	Claassens et al. 2023	LMG 31415; SARCC 753	8,276,659	63.99	7625	PRJNA680005	SAMN16869375	GCA_027594865
3925	type strain	*Bradyrhizobium yuanmingense*	CCBAU 10071	Yao et al. 2002	CIP 108027; NBRC 100594; CFNEB 101	8,201,482	63.77	7776	PRJEB14983	SAMN04487810	GCA_900094575
8842	type strain	*'Bradyrhizobium zhanjiangense'*	CCBAU 51778	Li et al. 2019	HAMBI 3648; LMG 29279	9,342,022	62.94	8857	PRJNA224116	SAMN03459138	GCF_004114935
115486	type strain	*Bradyrhizobium zhengyangense*	WYCCWR 13023	Zhang et al. 2023	GDMCC 1.3180; HAMBI 3760	8,997,574	62.87	8312	PRJNA797113	SAMN24966686	GCA_022012485

Table S7 : Taxonomic identification based on the results of the pairwise comparison of genome sequences: digital DNA: DNA hybridization (dDDH) between the *Bradyrhizobium* genome sequences from present study versus type strains and closely related *Bradyrhizobium* strains.

	CFUMN1		CFUSD1		CFUW1		JHI2701		JHI2711		Cens1A		DW10.1		DW12.5		USDA3010		LVM105		Cham227	
**Strain name**	**a**	**b**	**a**	**b**	**a**	**b**	**a**	**b**	**a**	**b**	**a**	**b**	**a**	**b**	**a**	**b**	**a**	**b**	**a**	**b**	**a**	**b**
CFUMN1			97.3	0.05	23.5	0.21	23.1	0.21	23.1	0.2	69	0.24	63.5	0.1	21.2	1.23	49.1	0.06	23.5	0.17	61.9	0.01
CFUSD1	97.3	0.05			23.5	0.26	23.1	0.25	23.1	0.25	68.3	0.28	62.9	0.1	21.2	1.23	49.4	0.02	23.5	0.22	61.7	0.04
CFUW1	23.5	0.21	23.5	0.26			55.2	0.01	55.2	0.01	24	0.03	23.3	0.1	21.3	1.19	23.7	0.27	92.6	0.04	23.2	0.22
JHI2701	23.1	0.21	23.1	0.25	55.2	0.01			100	0	24.3	0.03	23.2	0.1	30.6	1.44	23.7	0.27	56	0.04	23.2	0.21
JHI2711	23.1	0.2	23.1	0.25	55.2	0.01	100	0			24.3	0.03	23.2	0.1	30.3	1.44	23.6	0.26	56	0.03	23.2	0.21
Cens1A	69	0.24	68.3	0.28	24	0.03	24.3	0.03	24.3	0.03			70.8	0.2	21.3	1.17	51.7	0.3	24	0.07	72.8	0.24
DW10.1	63.5	0.09	62.9	0.13	23.3	0.13	23.2	0.12	23.2	0.12	70.8	0.15			21.3	1.32	50.6	0.15	23.4	0.08	67.4	0.09
DW12.5	21.2	1.23	21.2	1.23	21.3	1.19	30.6	1.44	30.3	1.44	21.3	1.17	21.3	1.3			31.1	1.21	30.3	1.44	20.8	1.23
USDA3010	49.1	0.06	49.4	0.02	23.7	0.27	23.7	0.27	23.6	0.26	51.7	0.3	50.6	0.2	31.1	1.21			23.7	0.23	52	0.06
LVM 105	23.5	0.17	23.5	0.22	92.6	0.04	56	0.04	56	0.03	24	0.07	23.4	0.1	30.3	1.44	23.7	0.23			23.1	0.18
**Cham227**	61.9	0.01	61.7	0.04	23.2	0.22	23.2	0.21	23.2	0.21	72.8	0.24	67.4	0.1	20.8	1.23	52	0.06	23.1	0.18		
B. erythrophlei CCBAU53325	33.1	0.3	33	0.25	22.6	0.51	22.8	0.5	22.8	0.5	34.7	0.54	33.9	0.4	21.1	0.93	34.1	0.24	22.5	0.47	32.9	0.29
***B . brasilense* B51278**	69.2	0.26	68.5	0.31	24	0.05	24.3	0.06	24.3	0.06	100	0.03	71	0.2	21.5	1.5	51.8	0.32	24	0.09	73	0.27
Bradyrhizobium sp. CCGUVB1N3	22.1	0.84	22	0.79	30.8	1.05	30.3	1.04	30.3	1.04	22.2	1.08	21.8	0.9	41	0.39	21.7	0.78	30.8	1.01	21.1	0.83
Bradyrhizobium sp. CCGUVB23	21.3	1.18	21.4	1.13	30.1	1.39	29.4	1.38	29.4	1.38	21.7	1.42	21.1	1.3	43	0.05	21	1.12	30	1.35	20.6	1.17
B . acaciae 10BB	50.3	0.02	50.6	0.02	23.4	0.23	23.7	0.23	23.7	0.22	53.3	0.26	54.4	0.1	20.9	1.21	59.6	0.04	23.4	0.19	54.1	0.02
B . agreste CNPSo 4010	23.2	0.09	23.2	0.14	49.9	0.12	59.8	0.11	59.8	0.11	24.2	0.14	23.2	0	30.4	1.33	23.3	0.15	50.1	0.08	23.5	0.1
B . altum Pear77	46.6	0.39	46.6	0.35	22.4	0.6	22.6	0.6	22.6	0.6	49.8	0.63	47.3	0.5	20.5	0.84	58.1	0.33	22.5	0.56	46.9	0.39
B. amphicarpaeae 39S1MB	23.8	0.94	23.7	0.99	50.5	0.73	50.9	0.74	50.8	0.74	24.8	0.71	24.3	0.9	31.5	2.18	23.8	1	51.3	0.77	23.3	0.95
B. arachidis LMG 26795	24.2	0.08	24.3	0.04	42.9	0.29	43.2	0.29	43.2	0.29	24.4	0.32	23.9	0.2	30.8	1.15	23.6	0.02	42.7	0.25	23.9	0.08
***B . australafricanum* WSM 4400**	63.9	0.19	64.4	0.15	23.1	0.4	23.2	0.4	23.2	0.4	71.6	0.43	65.7	0.3	21.2	1.04	49.3	0.13	23.2	0.36	74.9	0.19
B . barranii 144S4	23.5	0.59	23.4	0.54	36.8	0.8	36.2	0.79	36.1	0.79	22.8	0.83	22.1	0.7	28.2	0.64	22.7	0.53	36.5	0.76	21.8	0.58
B . barranii subsp. apii 38S5	23.6	0.37	23.5	0.32	36.7	0.58	36.6	0.58	36.6	0.57	23.5	0.61	22.6	0.5	29.3	0.86	23.4	0.31	36.6	0.54	22.2	0.36
B . betae PL7HG1	23.1	0.65	23	0.69	40.9	0.43	41.6	0.44	41.6	0.44	23.9	0.41	23.4	0.6	30.3	1.88	23.2	0.71	40.8	0.48	22.7	0.65
***B. brasilense* UFLA03-321**	64.6	0.22	64.7	0.26	24.1	0	24.2	0.01	24.2	0.01	75.6	0.02	75.9	0.1	21.5	1.45	54.8	0.28	24.1	0.05	78	0.22
B . cajani 1010	24.2	0.25	24.2	0.3	48.3	0.04	50.4	0.05	50.4	0.05	25	0.01	24.1	0.2	32.3	1.48	24.2	0.31	48.4	0.08	24	0.26
B . campsiandrae INPA 394B	21.9	0.06	22.1	0.02	37.7	0.27	37.3	0.27	37.3	0.27	22.4	0.3	22.3	0.2	29.2	1.17	22.4	0	37.6	0.23	21.6	0.06
B . centrolobi i BR 10245	22.6	0.49	22.6	0.45	34.8	0.71	34.6	0.7	34.6	0.7	23.6	0.73	22.7	0.6	31.5	0.74	22.3	0.43	34.6	0.66	22.5	0.49
B . centrosematis LMG 29515	24.2	0.34	24.1	0.38	52.9	0.13	52.6	0.13	52.5	0.14	25.2	0.1	24.5	0.3	31.4	1.57	23.6	0.4	52.9	0.17	23.7	0.34
B . commune BDV5040	23.4	0.21	23.2	0.25	38.9	0.01	39.4	0	39.4	0	24.3	0.03	23.8	0.1	31.7	1.44	23.3	0.27	39.3	0.04	23.2	0.21
B . cosmicum 58S1	23.2	0.62	23.2	0.67	45.4	0.41	45.5	0.41	45.5	0.42	24.2	0.38	23.7	0.5	30.9	1.85	23.9	0.68	45.4	0.45	22.9	0.63
B . cytisi CTAW11	23.3	0.54	23.2	0.5	36.6	0.75	36.9	0.75	36.9	0.75	23.7	0.78	23.4	0.6	31.3	0.69	23.3	0.48	36.7	0.71	22.8	0.54
B. daqingense CGMCC 1.10947	24.1	0.02	24	0.07	50	0.19	53.9	0.18	53.9	0.18	24.1	0.22	23.5	0.1	30.5	1.25	23.2	0.08	50.4	0.15	22.8	0.03
B. diazoefficiens USDA 110	25.6	0.35	25.5	0.39	46.6	0.13	44.9	0.14	44.9	0.14	24.7	0.11	24	0.3	32.1	1.58	24.3	0.41	46.7	0.18	23.5	0.35
B . diversitatis CNPSo 4019	22.7	0.21	22.6	0.26	48.3	0	50.4	0.01	50.4	0.01	23.6	0.03	23.2	0.1	29.1	1.44	22.5	0.27	49.4	0.04	22.4	0.22
***B. elkanii* USDA 76**	75.9	0	76.6	0.05	23.8	0.21	23.9	0.2	23.9	0.2	66.3	0.23	61.8	0.1	21.4	1.24	50.1	0.06	23.6	0.17	61.8	0.01
B. embrapense SEMIA 6208	48.4	0.26	48.4	0.31	24	0.05	24.7	0.06	24.7	0.06	52.1	0.03	51.8	0.2	21.2	1.49	64.4	0.32	23.9	0.09	51.6	0.27
B . erythrophlei CGMCC 1.13002	33.2	0.27	33.1	0.22	22.6	0.48	22.9	0.47	22.9	0.47	34.8	0.51	34	0.4	21.1	0.96	34.2	0.21	22.5	0.44	33	0.26
***B . ferriligni* CCBAU 51502**	70.3	0.35	70.7	0.3	23.2	0.56	23.2	0.55	23.2	0.55	68.7	0.59	64.7	0.4	21.3	0.88	50.7	0.29	23.3	0.52	63.3	0.34
B. forestalis INPA54B	23.6	0.15	23.6	0.2	53.9	0.06	55.4	0.05	55.3	0.05	24.6	0.08	23.7	0.1	30.4	1.38	23.7	0.21	54.7	0.02	23.5	0.16
***B. frederickii* CNPSo 3426**	23.5	0.13	23.6	0.18	83.7	0.08	55.3	0.07	55.2	0.07	24.2	0.1	23.6	0.1	31	1.37	23.9	0.2	85.2	0.04	23.3	0.14
B . glycinis CNPSo 4016	23	0.02	23	0.07	50.3	0.19	60.4	0.19	60.4	0.18	24	0.22	23.1	0.1	30.3	1.25	22.9	0.08	51	0.15	22.9	0.03
B . guangdongense CCBAU 51649	22.2	0.45	22.2	0.41	40.2	0.67	40.1	0.66	40.1	0.66	23	0.69	22.6	0.5	29.5	0.78	22.8	0.39	39.7	0.63	22.2	0.45
B . guangdongense CGMCC 1.15034	22.2	0.45	22.2	0.41	40.2	0.66	40.2	0.66	40.2	0.65	23	0.69	22.7	0.5	29.7	0.78	22.8	0.39	39.8	0.62	22.1	0.45
B. guangxiense CCBAU 53363	23.4	0.2	23.4	0.25	49.4	0.01	48.5	0.01	48.5	0	24.2	0.04	24	0.1	30.4	1.43	23.5	0.26	49.7	0.03	22.9	0.21
B. guangzhouense CCBAU 51670	22.2	0.32	22.2	0.27	39.1	0.53	39.5	0.53	39.5	0.52	23.1	0.56	22.7	0.4	30.7	0.91	22.6	0.26	39.1	0.49	21.9	0.31
B. huanghuaihaiense CGMCC 1.10948	25.2	0.23	25.1	0.27	45.6	0.01	47.1	0.02	47	0.02	24.6	0.01	24.2	0.1	31.3	1.46	23.6	0.29	45.8	0.06	23.5	0.23
B . iriomotense NBRC 102520	22.3	0.81	22.2	0.76	31.2	1.02	31.3	1.02	31.3	1.01	22.5	1.05	22.5	0.9	41.6	0.42	22	0.75	31.4	0.98	21.7	0.8
B. japonicum USDA 6	24.9	0.05	24.9	0	41.1	0.26	42.8	0.25	42.8	0.25	24.5	0.29	23.9	0.1	31	1.18	23.9	0.01	41.1	0.22	23.4	0.04
B . liaoningense NBRC 100396	23.2	0.24	23.4	0.28	47.3	0.03	48.9	0.03	48.9	0.03	23.1	0	22.6	0.2	29	1.47	22.9	0.3	47.3	0.07	22.1	0.24
B . manausense BR 3351	21.9	0.85	21.9	0.8	36.1	1.06	36.7	1.05	36.7	1.05	22.2	1.08	22.1	0.9	31.2	0.39	22	0.78	36	1.02	21.5	0.84
B. mercantei SEMIA 6399	47.6	0.28	47.2	0.32	23.1	0.07	23.1	0.07	23.1	0.07	51.2	0.04	52	0.2	21.6	1.51	48.8	0.34	23	0.11	48.1	0.28
B. nanningense CCBAU 53390	23.1	0.28	23.1	0.23	52.5	0.49	49.6	0.48	49.5	0.48	23.8	0.52	23.2	0.4	29.8	0.95	23.5	0.22	52.3	0.45	22.8	0.27
B. neotropicale BR 10247	23.2	0.11	23.3	0.07	37.1	0.33	37.1	0.32	37.1	0.32	24.1	0.35	23.4	0.2	33.4	1.12	23	0.05	37	0.29	22.8	0.11
B. niftali CNPSo 3448	23.5	0.18	23.5	0.14	44.6	0.39	44.4	0.39	44.4	0.39	24.1	0.42	23.5	0.3	31	1.05	23.9	0.12	44.6	0.35	23.5	0.18
B. nitroreducens TSA1	23.2	0.6	23.1	0.64	49.7	0.39	46.9	0.39	46.9	0.39	23.9	0.36	23.6	0.5	30.3	1.83	23.4	0.66	50.5	0.43	22.8	0.6
B . oropedii Pear76	47.7	0.14	47.5	0.1	22.8	0.35	23.2	0.35	23.2	0.35	51.4	0.38	49.2	0.2	20.7	1.09	54.5	0.08	22.7	0.31	48.5	0.14
B. ottawaense OO99	25.2	0.1	25.1	0.15	51.4	0.11	52.2	0.1	52.2	0.1	24.3	0.13	23.3	0	30.6	1.34	23.3	0.16	52	0.07	23.1	0.11
***B. pachyrhizi* PAC48**	66	0.05	66.2	0.09	23.6	0.16	24	0.16	24.1	0.16	75.7	0.19	65.6	0	21.1	1.28	50.6	0.11	23.4	0.12	65.9	0.05
B . quebecense 66S1MB	48.9	0.11	49.5	0.16	23.5	0.1	23	0.09	23	0.09	51	0.13	48.8	0	21.1	1.34	60.3	0.17	23.5	0.06	47.3	0.12
B . rifense CTAW71	22.4	0.69	22.5	0.65	34.7	0.9	35	0.9	35	0.9	23.1	0.93	22.6	0.8	30.1	0.54	23	0.63	34.7	0.86	22.3	0.69
B . sacchari P9-20	23.4	0.12	23.4	0.17	45	0.09	48.6	0.08	48.6	0.08	24.2	0.11	23.6	0	30.9	1.36	23.6	0.18	45.1	0.05	23.3	0.13
B . septentrionale 1S1	47.2	0.22	48	0.18	22.8	0.44	22.5	0.43	22.5	0.43	47.5	0.46	44.7	0.3	21.4	1.01	45.8	0.16	22.7	0.39	44.1	0.22
B. shewense ERR11	23.4	0.49	23.4	0.44	50.3	0.7	51.3	0.69	51.2	0.69	24.1	0.73	23.1	0.6	30.3	0.74	23.4	0.43	50.1	0.66	23.1	0.48
B. stylosanthis BR 446	23.6	0.84	23.6	0.89	43.6	0.63	44.2	0.64	44.2	0.64	24.4	0.6	24.1	0.76	31.9	2.07	23.8	0.9	43.8	0.67	23.3	0.85
B. symbiodeficiens 85S1MBT	23.2	0.56	23.2	0.6	50	0.35	49.5	0.35	49.5	0.35	24.3	0.32	23.8	0.47	31.1	1.79	23.5	0.62	51	0.39	23	0.56
B. tropiciagri SEMIA 6148	46.9	0.22	46.9	0.17	23.1	0.43	23	0.42	23	0.42	49.3	0.46	51.1	0.30	21	1.01	56.2	0.16	23	0.39	50.8	0.21
B. uaiense UFLA03-164	45.1	0.45	45.6	0.4	22.5	0.66	22.8	0.65	22.8	0.65	48.3	0.68	47.7	0.53	20.5	0.79	54.1	0.39	22.5	0.62	48.4	0.44
B. vignae LMG 28791	22.8	0.5	22.8	0.45	45	0.71	50.6	0.7	50.6	0.7	23.7	0.73	23.2	0.6	30.1	0.74	23.1	0.43	45.1	0.67	23.1	0.49
B. viridifuturi SEMIA 690	48.9	0.31	49.7	0.36	24	0.1	24.2	0.11	24.2	0.11	52.1	0.07	49.6	0.2	21.5	1.54	58.1	0.37	23.9	0.14	52.1	0.32
B . xenonodulans 14AB	23.5	0.28	23.5	0.32	44.9	0.06	45.5	0.07	45.5	0.07	24.8	0.04	24.4	0.2	32.5	1.51	24.3	0.34	44.8	0.1	23.5	0.28
***B. yuanmingense* CCBAU 10071**	22.9	0.06	23.2	0.1	51.3	0.15	79.3	0.15	79.3	0.15	23.6	0.18	22.9	0	29.6	1.29	23.1	0.12	52.6	0.11	22.3	0.06
B. zhanjiangense CCBAU 51778	23.7	0.77	23.7	0.73	46.4	0.98	47.7	0.98	47.7	0.98	23.9	1.01	23.2	0.9	30.7	0.46	23.4	0.71	46.5	0.94	23.4	0.77
B . zhengyangense WYCCWR 13023	21.7	0.84	21.7	0.8	36.9	1.05	37.2	1.05	37.2	1.05	22.3	1.08	22.1	0.9	30.5	0.39	22.3	0.78	36.9	1.01	21.6	0.84

[a] **dDDH (d0, in %)** Formula: 1 (HSP length / total length)

[b]**G+C content difference (in %)**

Probability that DDH > 70% (i.e., same species)

Probability that DDH > 79% (i.e., same subspecies)

Table S8a: OrthoFinder -overall comparative genomics statistics

Number of species	120
Number of genes	928796
**Number of genes in orthogroups**	912782
Number of unassigned genes	16014
Percentage of genes in orthogroups	98.3
Percentage of unassigned genes	1.7
**Number of orthogroups**	23879
Number of species-specific orthogroups	354
Number of genes in species-specific orthogroups	859
Percentage of genes in species-specific orthogroups	0.1
Mean orthogroup size	38.2
Median orthogroup size	8
G50 (assigned genes)	119
**G50 (all genes)**	118
O50 (assigned genes)	3078
O50 (all genes)	3146
**Number of orthogroups with all species present**	1369
Number of single-copy orthogroups	674

Table S8b: OrthoFinder: per species comparative genomics statistics

Name of strain	**Cens1A**	**CFUMN1**	**CFUSD1**	**CFUW1**	**DW10.1**	**DW12.5**	**JHI2701**	**JHI2711**	**USDA3010**	**LVM 105**	**Cham227**
Number of genes	7766	9022	9055	7758	8985	10642	7270	7270	8508	7538	8348
Number of genes in orthogroups	7694	8977	9009	7588	8425	9470	7269	7267	8340	7470	8230
Number of unassigned genes	72	45	46	170	560	1172	1	3	168	68	118
Percentage of genes in orthogroups	99.1	99.5	99.5	97.8	93.8	89	100	100	98	99.1	98.6
Percentage of unassigned genes	0.9	0.5	0.5	2.2	6.2	11	0	0	2	0.9	1.4
Number of orthogroups containing species	6659	7393	7347	6549	6991	7170	6474	6473	6919	6559	6724
Percentage of orthogroups containing species	27.9	31	30.8	27.4	29.3	30	27.1	27.1	29	27.5	28.2
Number of species-specific orthogroups	0	0	1	3	5	34	0	0	0	0	3
Number of genes in species-specific orthogroups	0	0	2	6	14	80	0	0	0	0	6
Percentage of genes in species-specific orthogroups	0	0	0	0.1	0.2	0.8	0	0	0	0	0.1

Table S9: Comparative analysis of nodulation genes (*nod* , *noe* and *nol* genes) that encode proteins involved in the biosynthesis and modification of Nod factors using EggNogg-Mapper and blastp+hmmer programme of PGPT-Pred module

Description	Gene	CFUMN1	CFUSD1	USDA 76	CCBAU 51502	CFUW1	LVM 105	CNPSo 3426	Cens1A	DW10.1	Cham227	WSM 4400	UFLA03-321	B51278	JHI2701	JHI2711	CCBAU 10071	USDA3010	66S1MB	DW12.5	NBRC 102520	Ec3.3	CCGUVB1N3	CCGUVB23	CGMCC	CCBAU 53325	CCGB01	CCGB12	CCGB20	CCGUVB14	CCGUVB4N	Tv2a-2	WSM2254	B51279	INPA54B
N-acyltransferase	***nodA***																																		
Chitooligosaccharide deacetylase	***nodB***																																		
N-acetylglucosaminyltransferase	***nodC***																																		
DNA-binding transcription factor activity (Transcriptional activator)	***nodD***																																		
Beta-ketoacyl synthase (adds C16 polyunsaturated fatty acid chain to Nod factor)	***nodE***																																		
Nod factor export ATP-binding protein I	***nodI***																																		
ABC transporter permease protein involved in secretion of Nod factors	***nodJ***																																		
6-O acteyl transferase at non-reducing end	***nodL***																																		
codes for D-glucosamine synthetase	***nodM***																																		
specific uridylyl transferase involved in the biosynthesis of lipopolysaccharide (LPS)	***nodN***																																		
encodes a Ca2(+)-binding protein that insert into lipid membranes and form ion channels, homologous to haemolysin	***nodO***																																		
SAM-utilizing methyltransferase involved in nod factor synthesis	***nodS***																																		
Outer membrane protein for secretion of Nod factors, Cultivar specific nodulation gene	***nodT***																																		
6-O- Carbamoyl transferase N-terminus	***nodU***																																		
a two-component regulatory system flavonoid sensor	***nodV***																																		
a two-component flavonoid dependent transcriptional activator protein	***nodW***																																		
PFAM acyltransferase 3 domain, Synthesis of Nod factor substituents	***nodX***																																		
Co-regulated with nodABC operon	***nodY***																																		
Fucosyl transferase essential for adding a fucose or 2-O-methylfucose residue to N-terminus	***nodZ***																																		
2-O-Methylation of Nod Factors	***noeI***																																		
Arabinosylation of the Nod factors	***noeC***																																		
Sulfotransferase, adds sulfate group to fucose sugar on Nod factor	***noeE***																																		
Transcriptional regulator -nol genes have several functions in synthesis of Nod factor substituents and their secretion	***nolA***																																		
Chitooligosaccharide deacetylase (cultivar specific)	***nolB***																																		
O-Acetyltransferase activity that modifies the fucose of Nod factor	***nolL***																																		
Nodulation protein nolO, is a carbamoyl transferase adding carbamoyl groups	***nolO***																																		
Global regulatory protein: transcriptional repressor	***nolR***																																		
Operon with nol (nolXWBTUV) cultivar specific nodulation	***nolU***																																		
Nodulation protein NolV (soybean cultivar specific) involved in the transfer of Nod factors	***nolV***																																		
NolY nodulation effectivity (genotype specific nodulation- *gsn* gene)	***nolY***																																		

***yellow filled cells indicates absence; green indicates presence of gene**
